# On-demand sIPN microneedles promote infected burn wound healing via microenvironment remodeling and activation of the Wnt–KLF5 regenerative axis

**DOI:** 10.7150/thno.130253

**Published:** 2026-05-01

**Authors:** Xiang Fang, Boquan Qin, Aaron Qi Zhang, Zhiyuan Zhang, Tingli Wang, Shizhou Wu, Hui Zhang, Hong Duan

**Affiliations:** 1 Department of Orthopedics, Orthopedic Research Institute, West China Hospital, Sichuan University, Chengdu, Sichuan, People’s Republic of China.; 2 Department of Anatomical and Cellular Pathology, State Key Laboratory of Translational Oncology, Li Ka Shing Institute of Health Sciences, Peter Hung Pain Research Institute, The Chinese University of Hong Kong, Hong Kong.; 3 National Engineering Research Center for Biomaterials, College of Biomedical Engineering, Sichuan University, Chengdu, Sichuan, People’s Republic of China.

**Keywords:** microneedles, infected burn wound, semi-interpenetrating polymer network, microenvironment remodeling, Wnt/KLF5 signaling

## Abstract

**Rationale:**

The healing of severe infected burn wounds is impeded by a vicious cycle of bacterial biofilms, oxidative stress, immune dysregulation, and hypoxia. Existing microneedle (MN) platforms often fail to address these multifactorial barriers due to insufficient mechanical robustness, lengthy fabrication times, and limited therapeutic scope. Our goal was to develop a multifunctional, on-demand MN platform that can simultaneously overcome these challenges by systematically dismantling pathological barriers and activating endogenous regenerative pathways.

**Methods:**

We developed a multifunctional MN platform based on a semi-Interpenetrating Polymer Network (sIPN) of hyaluronic acid methacrylate (HAMA) and ethoxylated trimethylolpropane triacrylate (ETPTA). This platform was co-encapsulated with a triad of therapeutic agents: a biofilm-dismantling antisense oligonucleotide (ASO) targeting the bacterial gene *yycF*, nanoceria (CeO₂) for reactive oxygen species (ROS) scavenging and in-situ oxygen generation, and anthocyanin (An) as an antioxidant and anti-inflammatory agent. The therapeutic efficacy of the MN platform was evaluated in a rat model of MRSA-infected full-thickness burns. Healing was assessed through macroscopic observation, histological analysis, and immunofluorescence staining. The underlying molecular mechanisms were investigated using transcriptomic and protein analyses of wound tissues.

**Results:**

The fabricated sIPN MNs exhibited exceptional mechanical strength, rapid fabrication time, and strong tissue adhesion. In the rat model, the MNs effectively dismantled biofilms, reduced oxidative stress, alleviated hypoxia, and shifted the immune balance towards M2 macrophage polarization. This comprehensive microenvironment remodeling led to accelerated wound closure, promoted angiogenesis, and encouraged ordered collagen deposition, resulting in higher-quality tissue regeneration compared to control groups. Transcriptomic and protein analyses revealed that this enhanced healing was driven by the significant activation of the epidermal Wnt/KLF5 signaling axis.

**Conclusions:**

Our study presents a mechanistically elucidated, multimodal sIPN MN platform that effectively promotes the healing of infected burn wounds. By remodeling the pathological microenvironment and activating the Wnt/KLF5 regenerative axis, this on-demand platform demonstrates significant potential for clinical translation in the management of complex wounds.

## Introduction

Severe burn injury is a serious global health challenge, triggering a complex cascade of pathophysiological events with implications far beyond the initial tissue damage 1, 2. Disruption of the skin barrier renders wounds susceptible to microbial invasion, often leading to the formation of persistent, drug-resistant bacterial biofilms 3. This infectious colonization occurs within a hostile wound microenvironment characterized by excessive oxidative stress caused by reactive oxygen species (ROS), a dysregulated immune microenvironment, severe tissue hypoxia, and impaired angiogenesis, creating a vicious cycle 4. This cycle not only impairs the host's innate repair mechanisms but also provides a harbour for pathogens, rendering conventional therapies largely ineffective. Consequently, many burn wounds deteriorate into a chronic, non-healing state, posing a significant risk of sepsis, increasing patient morbidity, and placing a significant burden on healthcare systems 5, 6. To overcome these challenges, therapeutic strategies must evolve from single-target interventions to integrated platforms that can simultaneously dismantle biofilms and actively reshape the pathological microenvironment to favor healing.

Microneedle arrays (MNs) have emerged as a promising strategy for treating complex skin wounds 7-10. Beyond cutaneous applications, MN-enabled delivery concepts have also been extended to other barrier tissues, highlighting the versatility of MN platforms for overcoming physiological transport limitations 11. MNs physically overcome major barriers to effective topical drug delivery by creating microchannels within the dense exopolysaccharide matrix of the stratum corneum and, crucially, bacterial biofilms 12. This allows drugs to be delivered directly to the deep, infected wound bed, where therapeutic intervention is most needed. However, despite these advances, the full therapeutic potential of MN technology in treating severe burns remains constrained by critical hurdles. Mechanically, the hard, tough nature of burn eschars requires exceptional penetration strength. Recent advances in bioinspired structural proteins and mechanically reinforced wearable biomaterials further underscore the importance of combining robustness with functional integration for next-generation microneedle-based devices 13, 14. Functionally, while current platforms excel at drug delivery, most focus on single-target interventions or distinct healing stages. Addressing the complex, multifactorial pathology of infected burns requires a more sophisticated capability to orchestrate a simultaneous, multi-stage synergistic attack—a level of integration that remains rare in existing designs. These functional challenges are further compounded by fundamental material limitations, particularly in conventional hydrogel systems such as those based on HAMA 15. While widely adopted for their biocompatibility, these matrices are inherently absorbable. In the context of moist wound management, this biodegradability transforms from an advantage into a critical liability. The matrix's rapid dissolution upon contact with exudate instigates a simultaneous dual failure: it triggers an uncontrolled "burst release" of the therapeutic payload—preventing sustained modulation—and concurrently obliterates the mechanical integrity required for long-term tissue anchorage. Beyond these performance issues, manufacturability remains a significant bottleneck; existing methods are often complex and time-consuming—typically requiring 24 to 48 hours for drying and demolding—which further limits their potential for rapid, on-demand clinical application 16, 17. Driven by the urgent need to overcome these combined mechanical, functional, and logistical hurdles, the present study develops an advanced MN platform characterized by rapid fabrication, superior mechanical robustness, and crucial structural integrity. Such a platform acts as a persistent device capable of being loaded with a complex, multifunctional therapeutic cargo to truly orchestrate the healing cascade.

**Figure 1:**
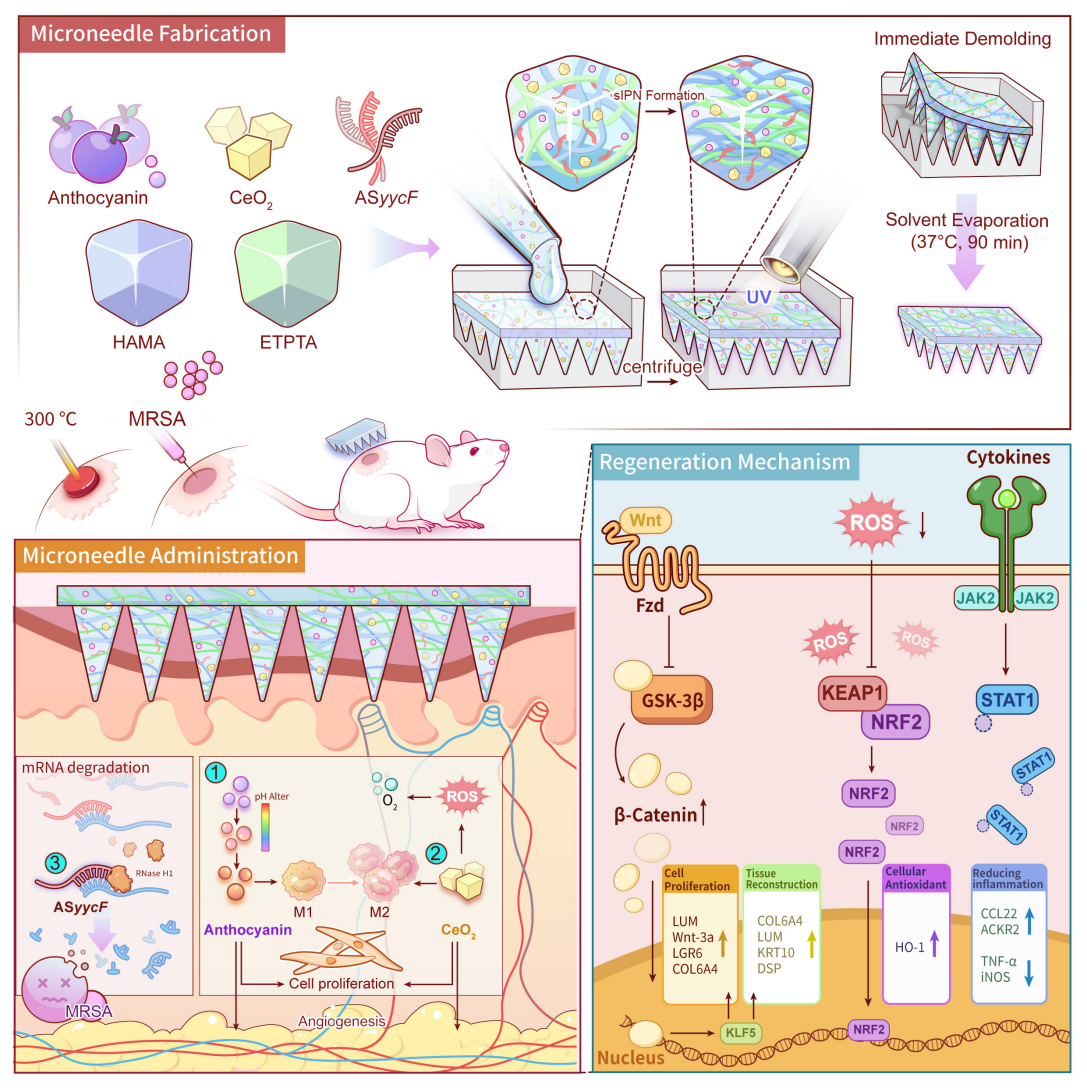
** Schematic illustration of the fabrication, application, and therapeutic mechanisms of the multifunctional MNs for infected burn wound healing.** The fabrication process involves the one-step encapsulation of An, CeO_2_, and AS*yycF* within a HAMA/ETPTA composite matrix, followed by UV polymerization and immediate demolding. Upon application to a MRSA-infected burn wound, the MNs patch delivers its therapeutic cargo to simultaneously eradicate bacteria and modulate the wound microenvironment. These coordinated actions activate the pro-regenerative Wnt/KLF5 signaling pathway, ultimately accelerating high-quality tissue regeneration.

Herein, we report an innovative, on-demand multifunctional MN platform built upon a unique semi-Interpenetrating Polymer Network (sIPN) matrix. This sIPN is fabricated through precise solvent engineering, which ensures the molecular-level integration of a synthetic monomer (ETPTA) and a bioactive polymer (HAMA). A subsequent, single-step photopolymerization forms the rigid, covalently crosslinked ETPTA network, which serves as the MNs' mechanical “backbone”, providing the requisite strength for eschar penetration. Simultaneously, the linear HAMA polymer chains become physically and stably entangled within this framework, functioning as both a bioactive component and a toughening agent. Crucially, this robust and persistent sIPN architecture, enabled by the rapid one-step fabrication, serves as an ideal vehicle for the simultaneous co-encapsulation of a multi-pronged therapeutic cargo. Upon application, the robust MNs physically penetrate the biofilm to deliver a customized ASO. Innovatively designed to circumvent antibiotic resistance by targeting the essential YycFG pathway, the *ASyycF* silences the critical transcription factor YycF, establishing a targeted gene silencing mechanism that effectively dismantles biofilm integrity 18-21. Concurrently, the MNs actively remodel the hostile microenvironment, wherein a dual-action system of anthocyanin (An) and nanoceria (CeO_2_) neutralizes the severe ROS storm and the CeO_2_ generates in-situ oxygen to combat tissue hypoxia 22, 23, while the pH-responsive nature of An may provide complementary real-time diagnostic feedback on the wound's pH status 24, 25. Collectively, by systematically dismantling these key pathological barriers, the MNs re-establishes a pro-regenerative microenvironment to accelerate the regeneration of infected burn wounds (Fig. 1).

## Methods

### Fabrication of MNs

The MNs arrays were fabricated using a photopolymerization method. First, a precursor solution for the H/E group was prepared. ETPTA (T837234, Macklin, China) was mixed with absolute ethanol at a 1:2 (v/v) ratio to form the ETPTA solution. A 2% (w/v) Hyaluronic Acid Methacrylate (HAMA-400K, EFL, China) solution was prepared using a 0.25% (w/v) LAP solution as the solvent. The final H/E precursor solution was obtained by mixing the HAMA solution and the ETPTA solution at a volume ratio of 2:3, followed by centrifugation at 2000 rpm for 2 minutes to remove bubbles, with the supernatant being collected for use.

For the therapeutic groups, active agents were incorporated into the H/E precursor supernatant. The concentrations of AS*yycF* and Anthocyanin were optimized based on previous studies to ensure therapeutic efficacy while minimizing potential cytotoxicity 18, 26, 27.

- For the **H/E@A** group, a 2% (w/v) An solution (C885847, Macklin, China) was added to the supernatant at a volume ratio of 1:12(v/v);
- For the **H/E@C** group, CeO₂, (104807, XFNANO, China) was added at a mass-to-volume ratio of 1:120(v/v);
- For the **H/E@ACA** group, both the An solution and CeO₂ were added, along with AS*yycF* (500 ng/μL) at a volume ratio of 1:120(v/v).

After thorough mixing, each resulting solution was cast into a polydimethylsiloxane (PDMS) MNs mold (Model TM-7, Henan Micro-Nano BenTeng Biotechnology Co., Ltd. China). The mold was centrifuged to fill the needle tips, topped up with additional solution, and then photopolymerized using UV light (365 nm) for 60 seconds. The MNs patches were immediately demolded and placed in an incubator at 37°C for 90 minutes to evaporate the solvent. Finally, the patches were sterilized with UV irradiation before use.

### Morphology of MNs

The gross appearance of the fabricated MNs patches was captured with a digital camera. The microstructure, including the morphology and dimensions of the individual microneedles, was examined using a Scanning Electron Microscope (SEM, ZEISS, German). To visualize the distribution of components within the MNs array, fluorescently labeled HAMA (green), ETPTA (blue), and An (red) were used. The spatial distribution was imaged and reconstructed into a 3D model using the confocal microscope (Andor, Austria). Furthermore, the elemental distribution of Cerium within the microneedles was characterized using Energy Dispersive Spectroscopy (EDS) mapping coupled with the SEM.

### Mechanical strength assessment

The mechanical robustness of the H/E@ACA MNs patches was evaluated by applying a series of calibrated weights (10 g, 20 g, 50 g, 100 g, 200 g, 500 g, and 1 kg) directly onto the needle tips to test their resistance to compressive forces.

### Swelling behavior

For the swelling assessment, disc-shaped constructs were prepared by photopolymerizing the H/E@ACA precursor solution. The constructs were lyophilized to obtain its dry weight (Wd). They were then immersed in PBS or Simulated Wound Fluid (SWF). The SWF was prepared by supplementing PBS with 30 mg/mL Bovine Serum Albumin (BSA) and 100 U/mL Collagenase to mimic the protein-rich and enzymatic character of wound exudate. At specific time points (4, 8, 12, 24, 48, 72, and 120 hours), the swollen constructs were removed, blotted to remove excess surface water, and weighed (Ws). The swelling ratio was calculated as (Ws - Wd) / Wd × 100%.

### *In vitro* drug release profile

Lyophilized H/E@ACA constructs were immersed in either PBS or SWF at 37°C with gentle shaking. At predetermined time points (4, 8, 12, 24, 48, 72, and 120 hours), the supernatant was collected for analysis, and an equal volume of fresh medium (either PBS or SWF) was replenished to maintain sink conditions. The cumulative release of An was quantified by measuring its absorbance using a UV-Vis-NIR spectrophotometer (Perkin-Elmer, USA). The concentration of released CeO2 was measured using Nanoparticle Tracking Analysis (Malven, UK). The release of AS*yycF* was quantified using a NanoDrop spectrophotometer (Thermo, USA).

### pH-responsive properties of An

The pH-sensitive chromic property of the encapsulated An was evaluated by immersing the fabricated H/E@ACA MN in buffer solutions with varying pH values (pH 4.0, 7.0, and 9.0). Following immersion, the samples were allowed to dry naturally to concentrate the released anthocyanin. The formation of colored halos around the base of the MN and their corresponding pH-dependent color shifts were visually documented.

### ROS scavenging assay

The H_2_O_2_ scavenging ability of the MNs components was assessed qualitatively. 200 μL of each solution (H/E, H/E@A, H/E@C, H/E@ACA) was mixed with 10 μL of 5% H_2_O_2_. After 1 minute of gentle shaking, the remaining H_2_O_2_ concentration was measured using a peroxide test strip (RUNBO®, China) with changes in color indicating the reduction of H_2_O_2_.

### Enzyme-mimetic activity assays

The catalase (CAT)- and superoxide dismutase (SOD)-like activities of the functionalized microneedles were evaluated using assay kits along with UV-Vis spectrophotometer. The absorbance values were recorded every 1 min up to 4 min. Specifically, the CAT-mimetic activity was determined by tracking the absorbance decrease at 240 nm, while the SOD-mimetic activity was assessed by measuring the absorbance change at 560 nm.

### Oxygen generation assay

To evaluate the sustained oxygen-generating capacity, 200 μL of each solution (Control, H/E, H/E@A, H/E@C, H/E@ACA) was mixed with 10 μL of 5% H₂O₂ in a sealed vial. A dissolved oxygen probe (JPBJ-608, INESA, China) was immediately immersed in the solution to continuously monitor the dissolved oxygen concentration every 5 minutes for a total duration of 60 minutes.

### Characterization of H/E@ACA composite

The particle size and zeta potential of the CeO_2_ were measured using Laser Mastersizer (Malven, UK). The chemical composition was analyzed using FTIR spectroscopy (Thermo, USA). Spectra were recorded from 4000–400 cm⁻¹ for three lyophilized samples: the H/E matrix, and the individually photopolymerized HAMA and ETPTA, which served as references.

### Preparation of extracts

Extracts of the materials were prepared in accordance with the ISO 10993-12 standard for all cellular experiments. Briefly, sterilized disc-shaped specimens from each group (H/E, H/E@A, H/E@C, and H/E@ACA) were immersed in cell culture medium at a ratio of 0.1 g/mL. The immersion was carried out at 37℃ for 24 h. Following incubation, the medium, hereafter referred to as the extract, was collected and sterilized by filtration through a 0.22μm syringe filter for subsequent experiments.

### *In vitro* biocompatibility assay

NIH/3T3 fibroblasts, HaCaT keratinocytes, and RAW 264.7 macrophages were seeded in a 24-well plate at a density of approximately 10,000 cells per well. The culture medium was then replaced with the prepared extracts. After 1, 3, and 7 days of incubation, the cells were stained with a Live/Dead staining kit (Calcein-AM and Propidium Iodide) according to the manufacturer's protocol and visualized using a fluorescence microscope. The cell viability was quantified by calculating the percentage of live cells from the fluorescence images using ImageJ software.

### *In vitro* hemocompatibility assay

The hemocompatibility was evaluated using a hemolysis assay with human red blood cells. Briefly, diluted red blood cell suspension was incubated with the material extracts at 37 °C. Water and PBS were used as positive and negative controls, respectively. After centrifugation, the absorbance of the supernatant was measured to calculate the hemolysis rate according to the ASTM F756 standard.

### Immunofluorescence staining

HUVECs, RAW 264.7, and NIH/3T3 cells were independently cultured on coverslips in 24-well plates. Following 48-hour treatment with various extracts, cells were fixed in 4% paraformaldehyde, permeabilized with 0.1% Triton X-100, and blocked with 5% BSA. Overnight incubation at 4°C with primary antibodies targeting VEGF, iNOS, and CD206 was performed, followed by incubation with fluorophore-conjugated secondary antibodies and nuclear counterstaining with DAPI. Fluorescence images were acquired using a confocal laser scanning microscope.

### Western blot analysis

Total protein was extracted from treated HUVECs, RAW 264.7, and NIH/3T3 cells using RIPA lysis buffer. Protein concentrations were determined using a BCA assay. Equal amounts of protein were separated by SDS-PAGE and transferred to PVDF membranes. The membranes were blocked and then incubated overnight at 4°C with primary antibodies against VEGF, iNOS, CD206, COL I, COL III, and β-Actin. After incubation with HRP-conjugated secondary antibodies, the protein bands were visualized using an ECL detection system. Band intensities were semi-quantified using ImageJ software.

### Flow cytometry analysis

Following 48-hour extract treatment, RAW 264.7 cells were harvested, washed, and stained with fluorescent antibodies against CD86-PE, CD206-APC, and F4/80-FITC (BioLegend, USA). Flow cytometric analysis was subsequently performed to quantify M1/M2 macrophage polarization based on surface marker expression.

### Quantitative real-time PCR

Total RNA was extracted from treated cells using Trizol reagent. cDNA was synthesized using a reverse transcription kit. qRT-PCR was performed using a SYBR Green Master Mix on a real-time PCR system. The relative gene expression levels of *VEGF*, *iNOS*, *CD206*, *COL I*, and *COL III* were calculated using the 2⁻ΔΔCt method, with GAPDH serving as the internal reference gene.

### Tube formation assay

HUVECs were seeded onto a Matrigel-coated 96-well plate and incubated with different material extracts. The formation of capillary-like tube structures was observed and photographed using an inverted microscope. The number of nodes and total mesh area were quantified using ImageJ software.

### Scratch wound healing assay

HUVECs were grown to a confluent monolayer in a 6-well plate. A scratch was created using a sterile pipette tip. The cells were then washed and incubated with different material extracts. Images of the scratch area were captured at 0, 8, 24, and 36 hours. The wound closure area was measured using ImageJ software to assess cell migration ability.

### *In vitro* antibacterial activity

All antibacterial experiments, with the exception of the zone of inhibition assay, were performed against Methicillin-resistant Staphylococcus aureus (MRSA) using the material extracts prepared as described in Section** Preparation of extracts.**

- **Live/Dead staining:** MRSA suspension was treated with different material extracts for 24 hours. The bacteria were then stained using a Live/Dead BacLight Bacterial Viability Kit and observed under a fluorescence microscope. The percentage of dead bacteria was quantified using ImageJ software.
- **SEM of bacterial morphology:** MRSA was treated with the material extracts as described above. After treatment, the bacteria were fixed with 2.5% glutaraldehyde, dehydrated through a graded ethanol series, and sputter-coated with gold for SEM imaging.
- **Crystal violet biofilm assay:** MRSA was cultured in a 24-well plate for 24 hours to form biofilms. The biofilms were then treated with different material extracts for another 24 hours. After washing, the remaining biofilms were stained with 0.1% crystal violet, and the plates were photographed for visual assessment.
- **Zone of inhibition assay:** MRSA suspension was uniformly spread on an agar plate. Sterilized MNs patches from each group were gently placed on the surface of the agar. The plate was incubated at 37°C for 24 hours, after which the zones of inhibition around each MNs patch were observed and photographed.
- **Antibacterial gene expression (qRT-PCR):** MRSA was treated with different material extracts for 24 hours. Total bacterial RNA was extracted, and qRT-PCR was performed to analyze the relative expression levels of key virulence-associated genes, *codY* and *yycF*.

### Infected burn wound model

All animal procedures were approved by the West China Hospital Animal Ethics Committee (Approval No. 20250310005). A total of 75 male Sprague-Dawley rats (8 weeks old) were used. The rats were anesthetized, and their dorsal hair was shaved and removed. A full-thickness burn wound was created by applying a 1.0 cm diameter copper stamp, maintained at a constant temperature of 300°C by a soldering iron (200W, 320g), to the dorsal skin for 15 seconds. This protocol was optimized based on previous studies to ensure the formation of a consistent full-thickness burn model while minimizing excessive mortality^28^. After cooling, the wound was inoculated with 100 μL of MRSA suspension (2.5 × 10⁷ CFU/mL) to establish an infected burn model.

### *In vivo* treatment and evaluation

The rats were randomly divided into five groups (n=5 per group): Control (no treatment), H/E, H/E@A, H/E@C, and H/E@ACA. On day 3 post-injury, the eschar was surgically excised. The wounds were then treated with the corresponding MNs patches. A second round of eschar removal and MNs application was performed on day 6. The wound healing process was photographically documented. On days 8, 16, and 24, rats were euthanized by an overdose of pentobarbital sodium, and wound tissues were harvested for analyses.

### Histological analysis

Harvested wound tissues were fixed in 4% paraformaldehyde, embedded in paraffin, and sectioned. For general morphology and inflammation assessment, sections were stained with H&E. Bacterial colonization within the tissue was assessed by Gram staining. Collagen deposition was evaluated using Masson's trichrome staining and Sirius Red staining. The Sirius Red-stained sections were also viewed under a polarized light microscope to analyze collagen fiber types. For immunohistochemistry (IHC) and immunofluorescence (IF), sections were stained with primary antibodies against CK5/6, COL-I, COL-III, CD206, and iNOS, followed by appropriate secondary antibodies. Images were captured using a slide scanner (VS200, Olympus, Japan), and quantitative analyses were performed using ImageJ software.

### RNA sequencing analysis

To investigate the underlying molecular mechanisms, wound tissues were harvested from the Control and H/E@ACA groups (n=3 per group) on day 16 post-treatment. Total RNA was extracted, and sequencing libraries were prepared for transcriptome sequencing on an Illumina platform. After quality control and mapping, differential expression analysis was performed. Genes with a P-value < 0.05 and a |log₂(Fold Change)| > 1 were identified as differentially expressed genes (DEGs). Gene Ontology (GO), Kyoto Encyclopedia of Genes and Genomes (KEGG) pathway analysis, and Gene Set Enrichment Analysis (GSEA) were conducted to identify enriched biological functions and signaling pathways.

### *In vivo* pathway verification

To validate the role of key signaling pathways, a separate *in vivo* experiment was conducted using the infected burn wound rat model. The rats were divided into four groups: Control, H/E@ACA, H/E@ACA + IWR-1-endo (Wnt inhibitor, Selleck), and H/E@ACA + ML264 (KLF5 inhibitor, MCE). The inhibitor groups received local injections of the respective inhibitor alongside the H/E@ACA MNs treatment. On day 16 post-treatment, wound tissues were collected for Western blot analysis. Total protein was extracted and quantified. Equal amounts of protein were separated by SDS-PAGE and transferred to PVDF membranes. The membranes were incubated with primary antibodies against Wnt3a and KLF5, with β-Actin serving as a loading control. After incubation with HRP-conjugated secondary antibodies, protein bands were visualized and quantified.

Furthermore, to spatially evaluate the Wnt-mediated epidermal regeneration, an additional *in vivo* cohort using the same rat model was established. The rats were grouped into Control, H/E@ACA, and H/E@ACA + XAV-939 (Wnt inhibitor, Selleck). Wound tissues were collected on day 16 and sectioned for immunofluorescence. Briefly, the sections were incubated with primary antibodies against β-catenin and Involucrin, followed by fluorophore-conjugated secondary antibodies and DAPI nuclear counterstaining. Fluorescence images were then captured and quantitatively analyzed.

### Statistical analysis

All statistical analyses were performed using GraphPad Prism software (Version 9.4, GraphPad Software, San Diego, California USA), which was also used for generating all graphs. Quantitative data are presented as the mean ± standard deviation (SD) from at least three independent experiments unless otherwise specified. For comparisons between two groups, an unpaired, two-tailed Student's t-test was used. For comparisons involving three or more groups, one-way analysis of variance (ANOVA) was performed, followed by Tukey's multiple comparisons post-hoc test. For microscopy, images were analyzed using Fiji (Windows 64, v1.51, NIH). A P-value of less than 0.05 was considered statistically significant.

## Results

### Fabrication and characterization of MNs

**Figure 2:**
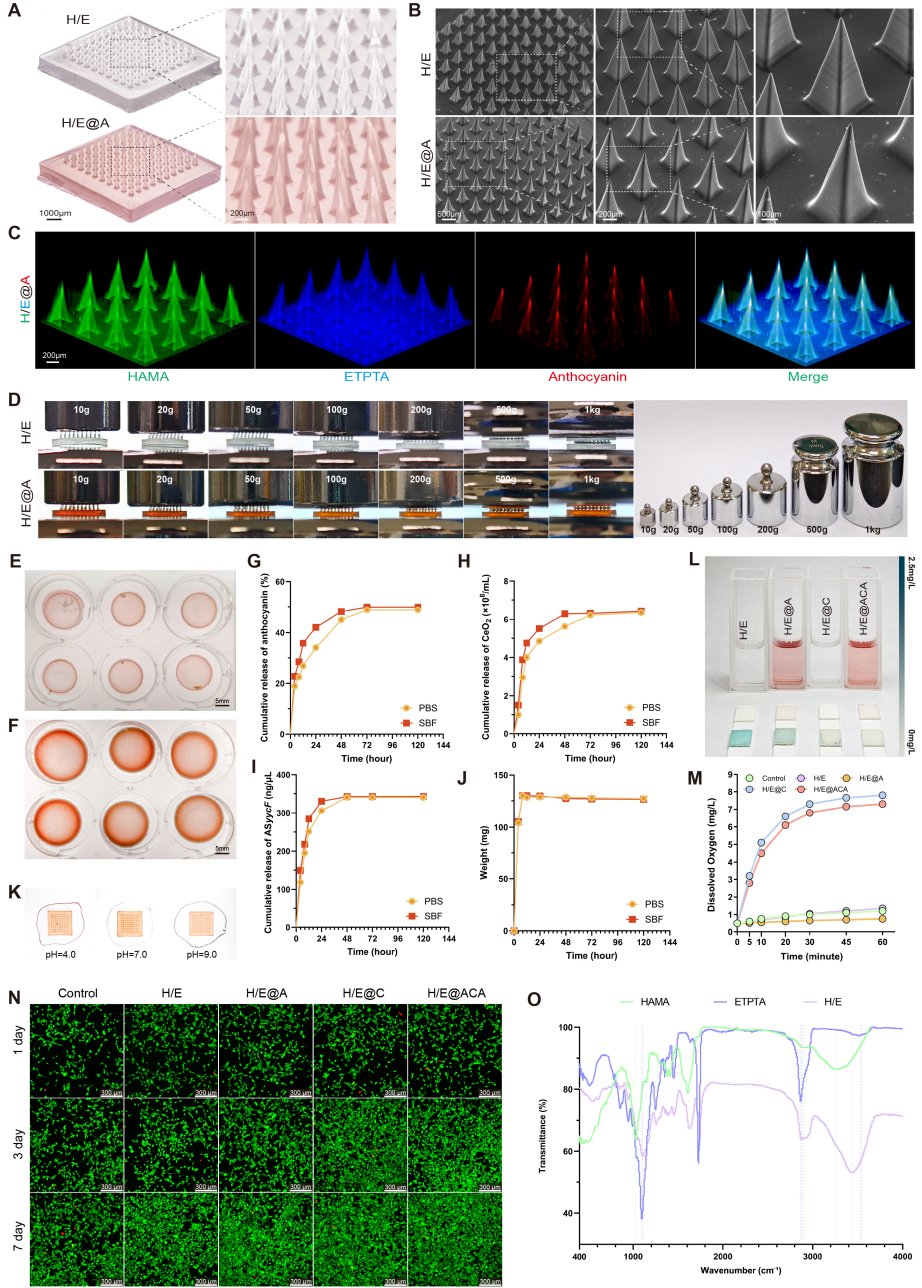
** Fabrication and physicochemical characterization of MNs.** (A) Gross photograph of a H/E@ACA MNs patch. (B) Scanning electron microscopy (SEM) images showing the morphology of the MNs array. (C) 3D fluorescence reconstruction of a single MNs visualizing the spatial distribution of HAMA (green), ETPTA (blue), and An (red). (D) Mechanical strength assessment under compressive loads. (E, F) Photographs showing the swelling behavior. (G-I) Cumulative release profiles of therapeutic agents: (G) An, (H) CeO2, and (I) AS*yycF*. (J) Swelling kinetics represented by weight change over time in PBS and SWF. (K) Visual demonstration of pH-responsive encapsulated anthocyanin release with the colored halo formation. (L) H₂O₂ scavenging assay. (M) Dynamic dissolved oxygen measurement assay.** (N)** Representative fluorescence images of Live/Dead staining of NIH/3T3 fibroblasts cultured with MNs extracts. (O) Fourier-transform infrared (FTIR) spectra of the lyophilized MNs formulations. Scale bars: 1000 μm, and 200 μm (from left to right in A); 500 μm, 200 μm, and 100 μm (from left to right in B); 200 μm (C); 5 mm (E, F); and 300 μm (N).

We successfully fabricated a multifunctional MNs array, designated H/E@ACA, designed for the comprehensive treatment of infected burn wounds. The MNs patch, composed of the HAMA and ETPTA composite matrix, appeared as a well-defined, translucent array with a reddish hue due to the encapsulated An (Fig. 2A). This successful fabrication is attributed to our efficient and reliable process. The inclusion of ETPTA provides immediate mechanical integrity after a 60-second UV photopolymerization, facilitating an instant, high-fidelity demolding process with a high success rate, as demonstrated in Supplementary Video 1. The resulting arrays consistently showed exceptional uniformity and structural integrity on a macroscopic level (Supplementary Fig. S1). SEM imaging confirmed the uniform formation of sharp, conical microneedles, approximately 550 μm in height and 250 μm in base diameter, which are ideal dimensions for penetrating the skin barrier and bacterial biofilms (Fig. 2B). To verify the successful co-encapsulation of multiple components, we performed 3D fluorescence reconstruction using fluorescently labeled materials. As shown in Fig. 2C, the structural matrix of HAMA (green) and ETPTA (blue) was homogenously integrated with An (red), confirming the successful one-step fabrication of the multi-agent delivery platform. Additionally, EDS mapping (Fig. S2) revealed a uniform spatial distribution of Cerium elements throughout the microneedle matrix, further verifying the successful and homogenous encapsulation of the nanoparticles.

The needles exhibited exceptional mechanical robustness, withstanding compressive loads up to 1 kg without structural failure, ensuring effective penetration of hardened eschar (Fig. 2D). Crucially, the patch also demonstrated strong adhesion to biological tissue, providing a stable interface for effective drug delivery, as shown in Supplementary Video 2. To evaluate their stability under physiological conditions, we assessed their behavior in both standard PBS and a more challenging SWF containing proteins and enzymes.

In PBS, the lyophilized MNs matrix showed significant swelling capacity, reaching a final swelling ratio of 127.12%, which facilitates drug release (Fig. 2E, F, J). The release kinetics of therapeutic agents followed a biphasic pattern, with an initial rapid release followed by a sustained phase. For instance, 27.20% of anthocyanin was released within the first 12 hours, ensuring immediate antioxidant activity (Fig. 2G). CeO₂ and AS*yycF* showed a similar sustained release profile (Fig. 2H, I). Notably, when challenged in the SWF, the constructs maintained excellent structural integrity and a comparable controlled release pattern. The swelling and release profiles in SWF closely mirrored those in PBS, confirming the robust stability of the sIPN matrix and its capability for reliable, long-term drug delivery even in a complex, physiologically relevant environment (Fig. 2G-J).

The encapsulated An retained its pH-responsive chromic properties, displaying a clear color shift from reddish-purple in acidic conditions to a darker blue-purple hue in alkaline conditions, where the formation of a colored halo around the MNs patch upon rehydration visually confirmed the successful release of the cargo into the surrounding medium (Fig. 2K). Furthermore, the MNs demonstrated potent ROS scavenging, with both CeO2 and An contributing to the rapid depletion of H₂O₂ (Fig. 2L). Quantitative kinetic assays further confirmed the robust CAT- and SOD-mimetic activities of the MNs (Fig. S3). Critically, real-time monitoring revealed that the CeO₂-containing groups generated a rapid surge of oxygen followed by a sustained high level of dissolved oxygen over 60 minutes, confirming the catalyst capabilities of the platform (Fig. 2M).

The constituent materials of the MNs were characterized to confirm their properties. The incorporated CeO₂ nanoparticles had a hydrodynamic diameter of approximately 150 nm and a zeta potential of -20 mV (Fig. S4). The Fourier-transform infrared (FTIR) spectroscopy provided key evidence for the sIPN structure (Fig. 2O). The HAMA spectrum showed its characteristic broad O-H absorption band (~3400 cm⁻¹) and amide I peak (~1650 cm⁻¹). The ETPTA was identified by its prominent ester carbonyl (C=O) peak at ~1730 cm⁻¹ and a sharp peak at ~810 cm⁻¹ from its vinyl (C=C) bonds. In the final H/E matrix, the complete disappearance of the vinyl peak at ~810 cm⁻¹ confirmed the successful photopolymerization of ETPTA. Critically, the H/E spectrum retained the characteristic peaks of both HAMA and ETPTA backbone. This confirmed the successful formation of the sIPN structure, as the spectrum of the final matrix was a physical superposition of the characteristic peaks of both HAMA and ETPTA without evidence of covalent cross-linking between them.

The biocompatibility of the MNs was evaluated using NIH/3T3 fibroblasts. Live/Dead staining revealed a high prevalence of viable cells when cultured with extracts from all material groups for up to 7 days (Fig. 2N). Quantitative analysis further confirmed that cell viability remained above 95% at all time points for all groups, demonstrating that the co-encapsulation of anthocyanin, CeO₂, and AS*yycF* did not induce cytotoxicity (Fig. S4). Consistently, supplementary assays extended to HaCaT keratinocytes and RAW 264.7 macrophages demonstrated similarly high viability, confirming the broad cytocompatibility of the platform (Fig. S5). Furthermore, the *in vitro* hemolysis assay demonstrated that the hemolysis rates for all groups were less than 5% (Fig. S6), well below the safety threshold defined by the ASTM F756 standard, confirming the excellent blood compatibility of the microneedles.

### *In vitro* antibacterial and pro-regenerative efficacy

**Figure 3:**
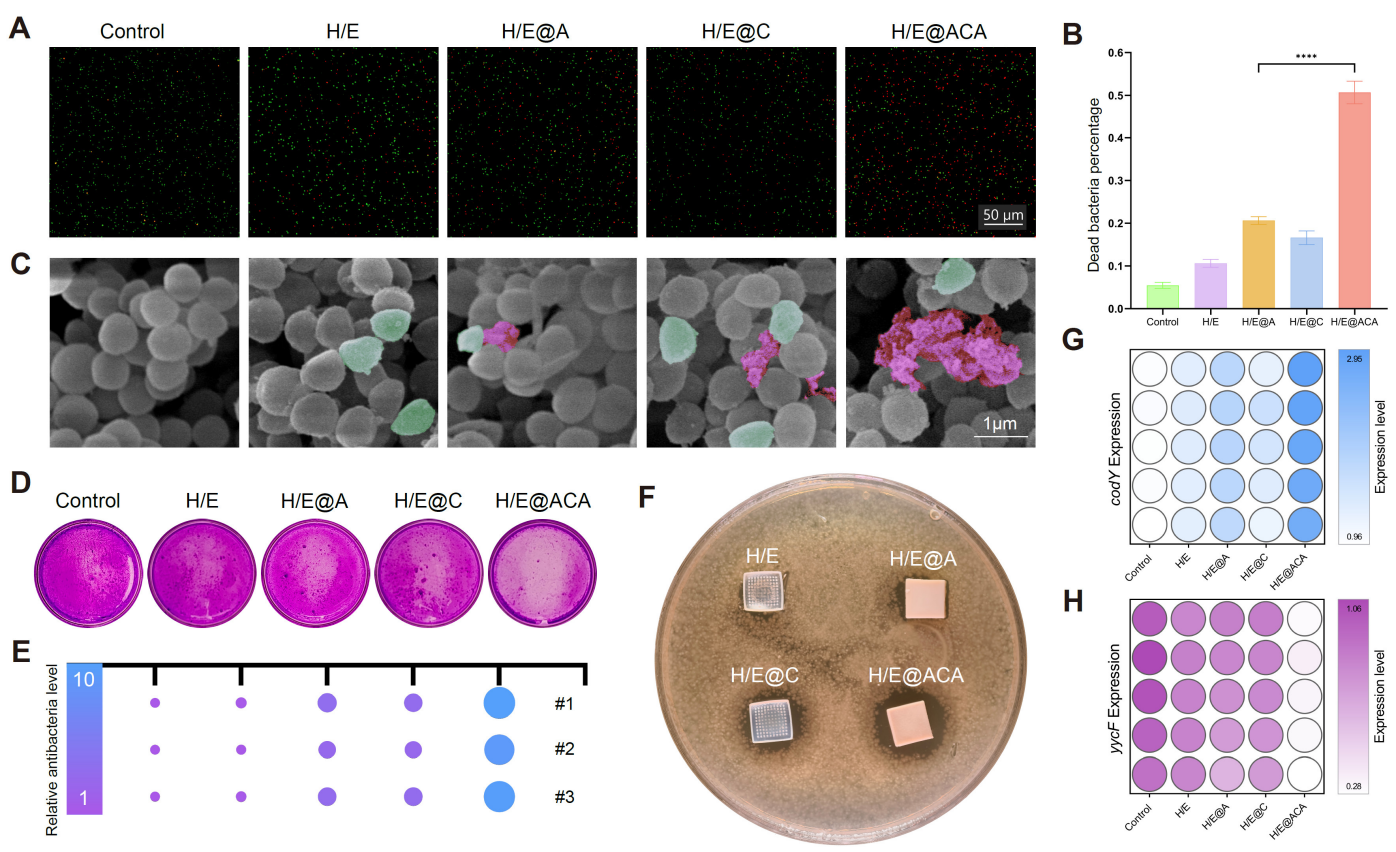
***In vitro* antibacterial efficacy of MNs.** (A, B) Live/Dead staining of MRSA after treatment with MNs extracts: (A) representative fluorescence images and (B) quantitative analysis. (C) SEM images of MRSA morphology after treatment with MNs extracts, with wrinkled bacteria in green pseudo-color and ruptured bacteria in red pseudo-color. (D, E) Crystal violet staining of MRSA biofilms after treatment with MNs extracts: (D) representative images and (E) corresponding visual representation. (F) Zone of inhibition assay with MNs patches. (G, H) qRT-PCR analysis of the relative expression of bacterial genes *codY* (G) and *yycF* (H) after treatment. Data are presented as mean ± SD. Statistical significance was analyzed using one-way ANOVA with Tukey's post-hoc test. ****p < 0.0001. Scale bars = 50 µm (A), and 1 µm (C).

To validate the antibacterial efficacy of the MNs, a series of *in vitro* experiments were conducted against MRSA. Live/Dead staining showed a substantial increase in the proportion of dead bacteria after treatment with H/E@ACA extract, with the percentage of dead cells exceeding 50%, significantly higher than all other groups (Fig. 3A, B). SEM imaging revealed the mechanism of action, where bacteria in the control group displayed smooth, intact surfaces, while those treated with H/E@ACA appeared shrunken, wrinkled, and even fragmented, indicating severe cell membrane damage (Fig. 3C). The H/E@ACA extract also demonstrated superior anti-biofilm capabilities. In the crystal violet assay, the H/E@ACA group showed visibly less biofilm biomass compared to other groups, indicating effective inhibition of biofilm formation or eradication of existing biofilms (Fig. 3D, E). The efficacy was further confirmed by the zone of inhibition test, where only the H/E@ACA MNs patch created a clear, bacteria-free halo on the agar plate, demonstrating potent antibacterial activity (Fig. 3F). At the molecular level, qRT-PCR analysis showed that H/E@ACA treatment significantly downregulated the expression of key bacterial virulence genes *codY* and *yycF* (Fig. 3G, H).

**Figure 4:**
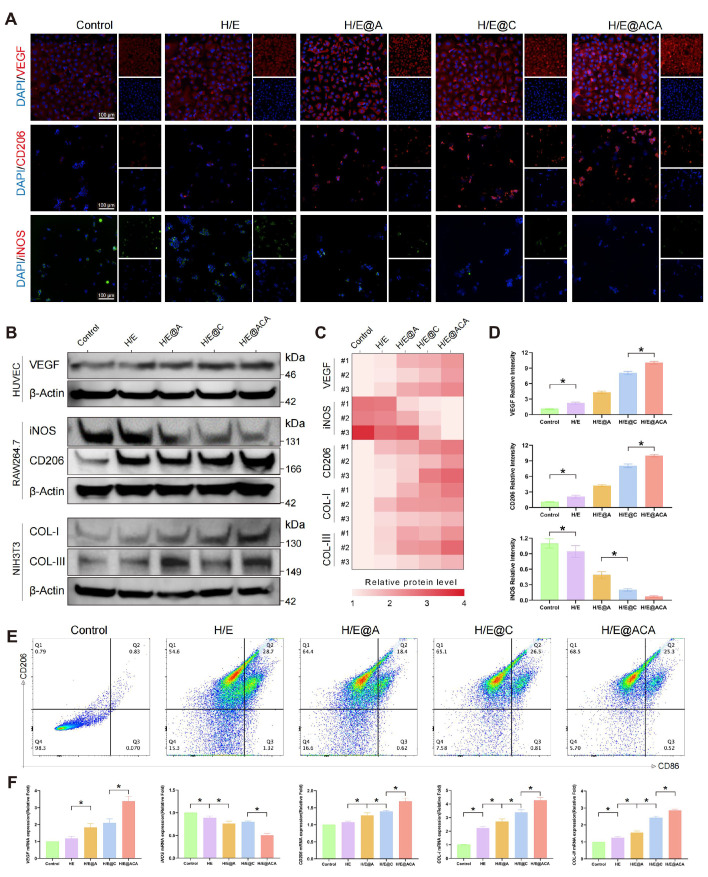
** MNs promote immunomodulation, angiogenesis, and collagen synthesis *in vitro*.** (A, D) Immunofluorescence staining of HUVECs and RAW 264.7 cultured with different MNs extracts: (A) representative images for VEGF, CD206, and iNOS and (D) corresponding quantitative analysis. Scale bar = 100 µm. (B, C) Western blot analysis of cell lysates after treatment with MNs extracts: (B) representative bands for key proteins in corresponding cell lines and (C) semi-quantitative analysis. (E) Flow cytometry analysis of the percentage of CD206-positive RAW 264.7 cells after treatment. (F) Quantitative real-time PCR (qRT-PCR) analysis of relative gene expression in cells after treatment. Data are presented as mean ± SD. Statistical significance was analyzed using one-way ANOVA with Tukey's post-hoc test. *p < 0.05. Scale bars = 100 µm (A).

Concurrently, we investigated the effects of the MNs extracts on key cell types involved in tissue repair. Immunofluorescence imaging of RAW 264.7 showed that the H/E@ACA group had an increased expression of the M2-phenotype marker CD206 and a decreased expression of the M1-phenotype marker iNOS when compared to the control group (Fig. 4A, D). This observation was corroborated at the protein level by Western blot (Fig. 4B, C), at the gene level by qRT-PCR (Fig. 4F), and through flow cytometry, which showed a higher percentage of CD206-positive cells in the H/E@ACA group (Fig. 4E). The MNs extract also influenced cells related to angiogenesis and collagen deposition. HUVEC treated with H/E@ACA extract showed upregulated expression of VEGF, as determined by Western blot and qRT-PCR (Fig. 4B, C, F). Similarly, NIH/3T3 fibroblasts treated with H/E@ACA extract exhibited increased expression of both COL I and COL III at both gene and protein levels (Fig. 4B, C, F). The angiogenesis function of MNs was further evaluated. In a scratch wound healing assay, HUVECs treated with H/E@ACA extract displayed faster wound closure compared to all other groups (Supplementary Fig. S7). This pro-migratory effect was complemented by results from a tube formation assay, where HUVECs cultured with H/E@ACA extract formed more extensive capillary-like networks, showing a greater number of nodes and a larger total mesh area compared to controls (Supplementary Fig. S7).

### MNs accelerate infected burn wound healing *in vivo*

**Figure 5:**
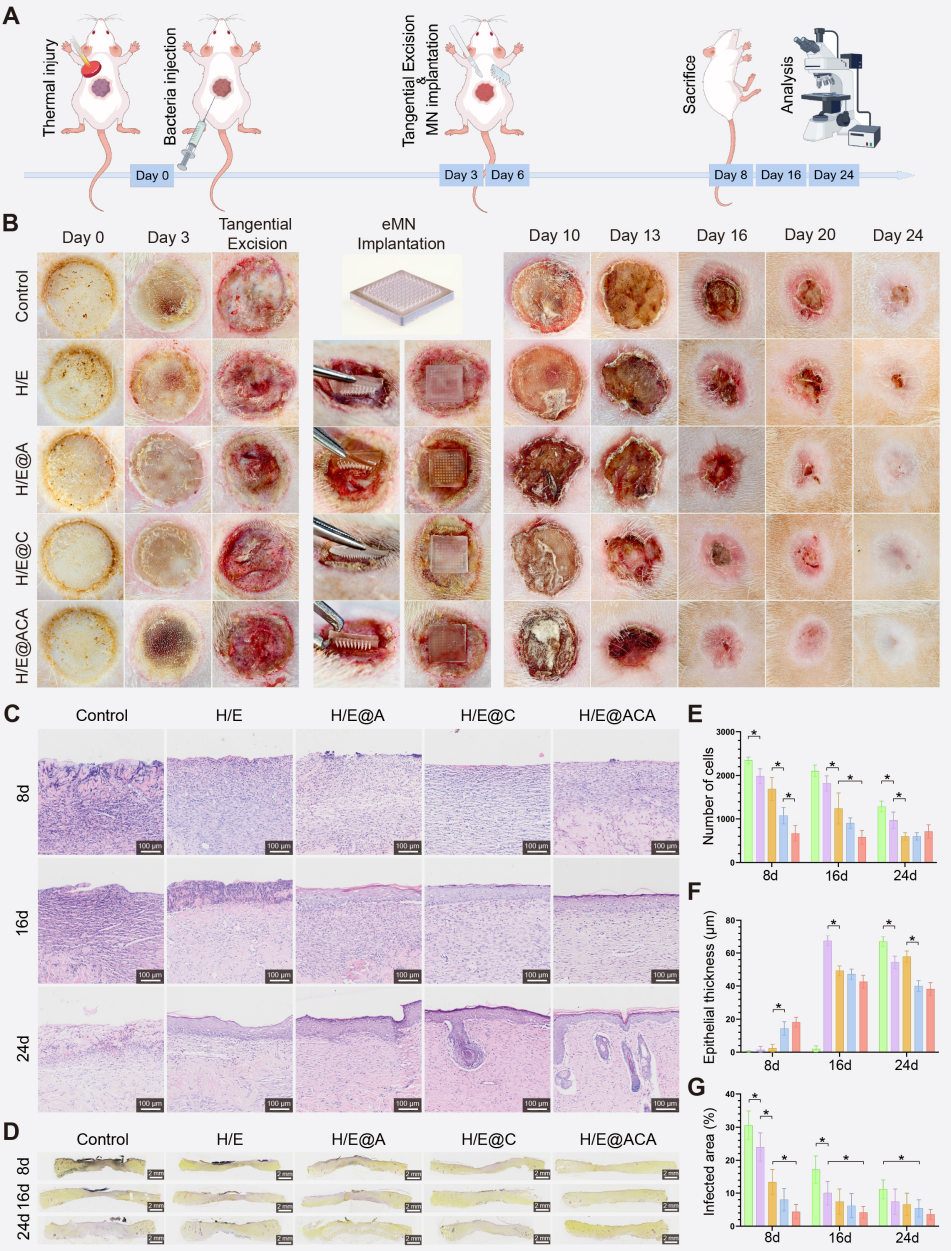
** Accelerated healing in a rat model of infected burn wounds.** (A) Schematic of the *in vivo* experimental design. (B) Macroscopic images of wounds at different time points. (C) Representative H&E staining images of wound tissues. (D) Representative Gram staining images of wound tissues. (E, F) Quantitative analysis of total number of cells and epithelial thickness. (G) Quantitative analysis of the infected area percentage. Data are presented as mean ± SD. Statistical significance was analyzed using one-way ANOVA with Tukey's post-hoc test. *p < 0.05. Scale bars = 100 µm (C), and 2 mm (D).

Encouraged by the *in vitro* results, we evaluated the therapeutic efficacy of the MNs in a clinically relevant rat model of MRSA-infected full-thickness burn wounds (Fig. 5A). Macroscopic observation of the wounds over 24 days revealed a markedly accelerated healing process in the H/E@ACA group, as quantified by the wound closure rates (Figure S8). While wounds in the control and H/E groups showed persistent inflammation and delayed closure, the H/E@ACA-treated wounds exhibited rapid granulation tissue formation and re-epithelialization, achieving near-complete closure by day 24 (Fig. 5B). Histological analysis confirmed these observations. H&E staining on day 8 showed extensive inflammatory cell infiltration and necrosis in the control group, whereas the H/E@ACA group displayed well-organized granulation tissue and early signs of re-epithelialization (Fig. 5C). Quantitative analysis showed a progressive decrease in epithelial thickness towards normal levels in the H/E@ACA group over time (Fig. 5E, F). Gram staining demonstrated a significant reduction in bacterial load within the wound bed of the H/E@ACA group at all time points, confirming the potent *in vivo* antibacterial effect (Fig. 5D, G).

**Figure 6:**
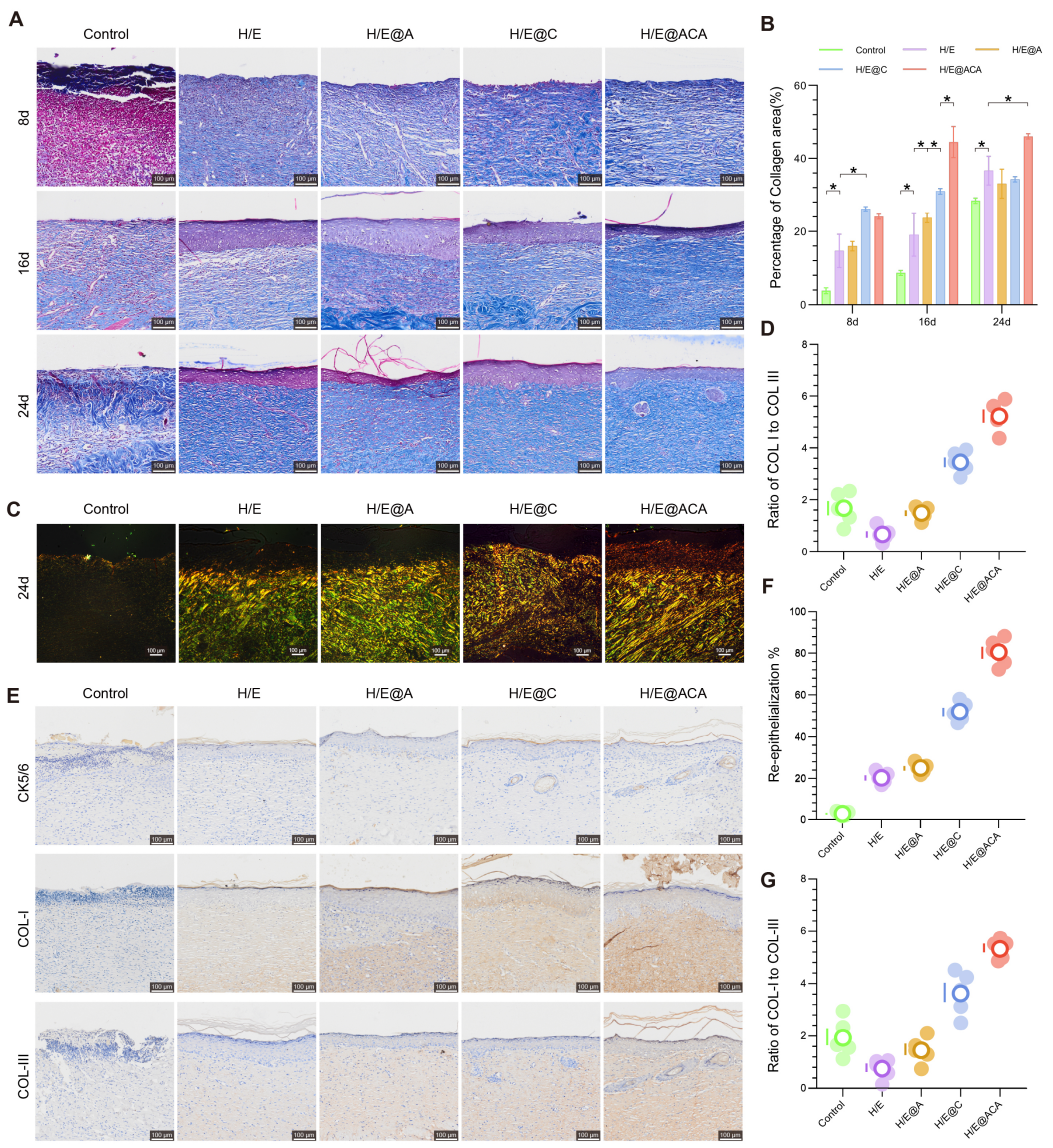
** Promotion of ECM deposition and skin regeneration.** (A, B) Masson's trichrome staining of wound tissues: (A) representative images and (B) corresponding quantitative analysis of the percentage of collagen area. (C, D) Polarized light microscopy of Masson-stained sections on day 24: (C) representative images and (D) corresponding quantitative analysis of the ratio of Type I to Type III collagen. (E) Representative immunohistochemistry (IHC) images for CK5/6, COL I, and COL III expression in wound tissues on day 24. (F) Quantitative analysis of the re-epithelialization rate from CK5/6 staining. (G) Quantitative analysis of the ratio of COL I to COL III from IHC staining. Data are presented as mean ± SD. Statistical significance was analyzed using one-way ANOVA with Tukey's post-hoc test. *p < 0.05. Scale bars = 100 µm (A, C, E).

We next assessed the quality of the regenerated tissue. Masson's trichrome staining showed that the H/E@ACA-treated wounds had a significantly higher percentage of collagen area at all time points, indicating robust extracellular matrix (ECM) deposition (Fig. 6A, B). To evaluate collagen maturity, which is crucial for scarless healing, we analyzed the ratio of Type I to Type III collagen using polarized light microscopy. On day 24, the H/E@ACA group exhibited a COL I/COL III ratio that was significantly lower than the control groups and closer to that of healthy skin, suggesting the formation of more regenerative, less fibrotic tissue (Fig. 6C, D). Immunohistochemical staining for CK5/6, a marker for basal keratinocytes, showed a significantly higher re-epithelialization rate in the H/E@ACA group, indicating faster and more complete epidermal barrier restoration (Fig. 6E, F). Staining for COL I and COL III further confirmed the ECM remodeling results, with the H/E@ACA group displaying a balanced deposition of both collagen types, ultimately leading to a favorable COL I/COL III ratio conducive to high-quality skin regeneration (Fig. 6E, G).

**Figure 7:**
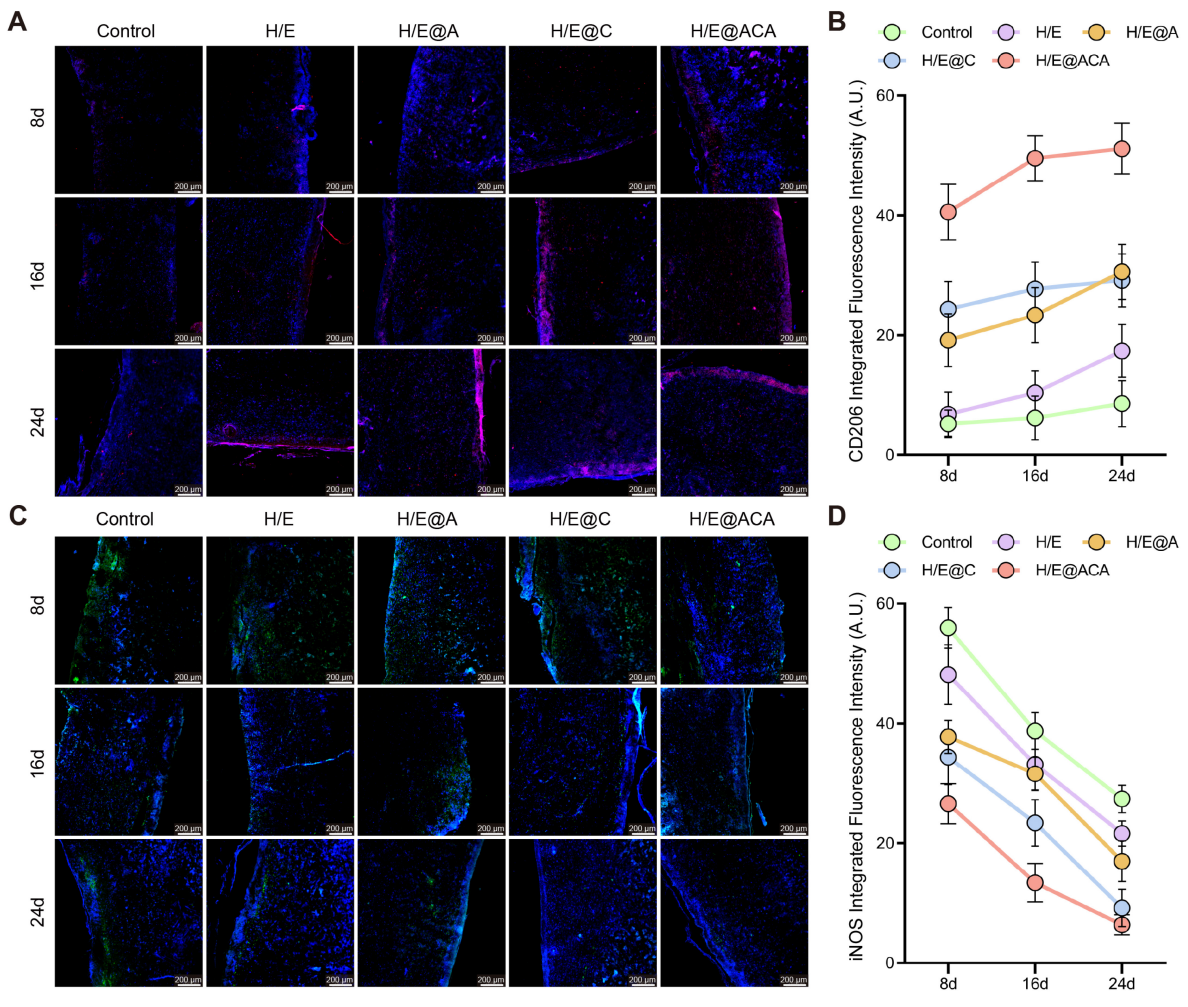
** Modulation of the *in vivo* inflammatory microenvironment.** (A, B) Immunofluorescence staining for CD206 (M2 marker) in wound tissues: (A) representative images and (B) quantitative analysis of integrated fluorescence intensity. (C, D) Immunofluorescence staining for iNOS (M1 marker): (C) representative images and (D) quantitative analysis of integrated fluorescence intensity. Data are presented as mean ± SD. Statistical significance was analyzed using one-way ANOVA with Tukey's post-hoc test. Scale bars = 200 µm (A, C).

The *in vivo* immunomodulatory effects were also investigated by tracking macrophage polarization within the wound tissue. Immunofluorescence staining revealed that H/E@ACA treatment led to a significant and sustained increase in the expression of the M2 macrophage marker CD206 from day 8 to day 24 (Fig. 7A, B). Conversely, the expression of the M1 macrophage marker iNOS was markedly suppressed in the H/E@ACA group throughout the healing process (Fig. 7C, D). This demonstrates a decisive shift in the wound's immune microenvironment from a chronic inflammatory state to a pro-regenerative state.

### MNs therapy activates the Wnt/KLF5 signaling pathway

**Figure 8:**
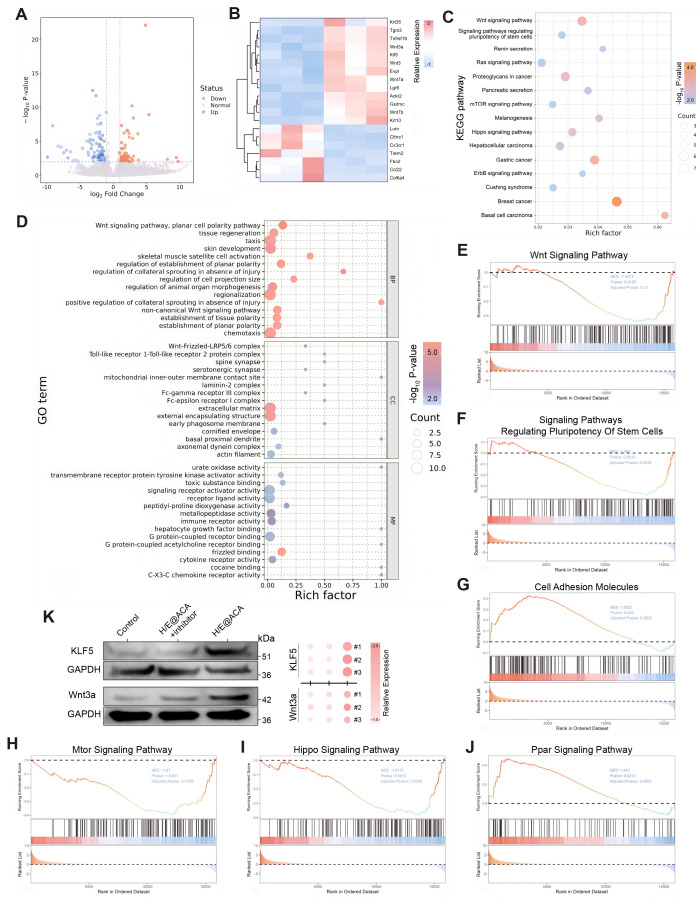
** RNA-seq analysis and pathway validation.** (A) Volcano plot of differentially expressed genes (DEGs). (B) Heatmap showing clustering of DEGs. (C) KEGG pathway enrichment analysis. (D) GO enrichment analysis. (E-J) GSEA plots for key signaling pathways. (K) Western blot validation showing the expression of Wnt3a and KLF5 in the Control, the H/E@ACA groups treated with their respective inhibitors (IWR-1-endo for the Wnt pathway; ML264 for KLF5), and the H/E@ACA group.

To gain deeper insight into the molecular mechanisms driving the enhanced healing, we performed RNA sequencing on wound tissues from the Control and H/E@ACA-treated rats. The treatment induced a significant transcriptomic shift, with numerous genes being differentially expressed between the two groups (Fig. 8A, B). KEGG pathway analysis of the upregulated genes further confirmed this pro-regenerative shift, showing significant enrichment in pathways such as the "Wnt signaling pathway", "Hippo signaling pathway", and "mTOR signaling pathway" (Fig. 8C). To understand the functional implications of these changes, we performed GO and GSEA. GO enrichment analysis of the upregulated genes revealed a highly coordinated functional synergy centered around cell communication and tissue remodeling (Fig. 8D). At the molecular function (MF) level, these genes were enriched in "receptor ligand activity," indicating a crucial role in initiating signal transduction. These molecular activities were spatially localized to the "extracellular matrix" and membrane-related complexes (Cellular Component, CC). Ultimately, these processes drove key biological processes (BP) such as "tissue regeneration" and "skin development." GSEA further elucidated the specific signaling pathways modulated by the H/E@ACA treatment. The analysis showed significant positive enrichment for several key pro-regenerative pathways (Fig. 8E-J). Notably, the "Wnt Signaling Pathway" was highly enriched, a cornerstone pathway in skin development and regeneration (Fig. 8E). We also observed enrichment in "Signaling Pathways Regulating Pluripotency of Stem Cells" (Fig. 8H), "mTOR signaling" (Fig. 8F), and "Hippo signaling" (Fig. 8I), collectively pointing to a multi-faceted activation of pro-healing molecular machinery.

Prompted by the transcriptomic findings, we performed a comprehensive *in vivo* experiment to validate the role of the Wnt signaling pathway and the transcription factor KLF5 at the protein level. Western blot analysis of wound tissues harvested on day 16 demonstrated that H/E@ACA treatment significantly upregulated the expression of both Wnt3a and KLF5 compared to the control group. Importantly, this upregulation was effectively reversed by the co-administration of their respective inhibitors; the Wnt pathway inhibitor IWR-1-endo abrogated the increase in Wnt3a, while the KLF5 inhibitor ML264 prevented the upregulation of KLF5 (Fig. 8K). To further validate the spatial activation of this pathway, immunofluorescence staining was performed. The results showed a specific upregulation and nuclear accumulation of β-catenin and increased expression of the differentiation marker Involucrin in the regenerating epidermis of the H/E@ACA group, which was abrogated by the specific inhibitors (Figure S9). These integrated results provide strong evidence that the H/E@ACA platform promotes wound healing by actively engaging the Wnt and KLF5 pro-regenerative signaling pathways.

## Discussion

The treatment of severe infected burn wounds presents a significant clinical challenge, primarily due to a complex pathological cascade involving persistent bacterial biofilms, severe oxidative stress, a dysregulated immune microenvironment, profound tissue hypoxia, and impaired angiogenesis, which collectively hinder the natural healing process 29. Addressing these interrelated issues requires a comprehensive therapeutic platform capable of simultaneous multi-pronged intervention. In this study, we successfully developed a multifunctional H/E@ACA MNs that was specifically engineered to overcome these challenges. Our MNs demonstrate a critical combination of features: on-demand fabrication, superior mechanical strength, and persistent tissue adhesion. As our results show, these advanced physical properties were essential for the MNs to stably anchor within the wound bed, thereby allowing its encapsulated therapeutic cargo to achieve comprehensive biofilm eradication and a thorough remodeling of the hostile microenvironment.

A key innovation of our work is the rational design of a sIPN matrix, which synergistically overcomes the inherent compromises of its individual components: conventional HAMA MNs are biocompatible but mechanically weak and rapidly dissolve, while ETPTA MNs are rigid but brittle and bio-inert. Our strategy utilized solvent engineering by using ethanol as a co-solvent to achieve a homogenous, molecular-level co-solution of the otherwise immiscible hydrophobic ETPTA and hydrophilic HAMA. A subsequent single-step photopolymerization formed the sIPN MNs. This structure was confirmed by FTIR (Fig. 2O), which showed the presence of both components without new covalent bonds, and by swelling assays (Fig. 2E-F), which demonstrated that the HAMA chains were physically entangled and constrained by the rigid ETPTA network.

This proven sIPN architecture yields a unique combination of performance advantages. The ETPTA 'skeletal' network provides the exceptional mechanical robustness required for eschar penetration (Fig. 2D). Simultaneously, the physically entrapped HAMA imparts biocompatibility and strong, dual-mode tissue adhesion (Supplementary Video 2) via physical hydrogen bonding and biological CD44 receptor binding 30, 31. Critically, the rigid ETPTA network provides a non-dissolving anchor for the HAMA chains, resolving the classic strength-versus-functionality trade-off. Furthermore, this composition enables immediate, high-fidelity demolding (Supplementary Video 1), facilitating a rapid (~90 min) on-demand fabrication protocol that stands in stark contrast to the 24–48 hours of drying and demolding required by conventional methods 32, 33 (Table S1). The resulting architecture demonstrated remarkable structural stability. This resilience was first validated by our *in vitro* studies, where the sIPN matrix maintained its structural integrity and controlled release function even when challenged with a SWF containing proteases. This was further characterized by a 'Dynamic Structural Reset': even after six months of immersion in PBS or culture medium, which confirmed their resistance to degradation, the microneedles reverted to a sharp morphology nearly identical to their as-fabricated state upon subsequent drying (Supplementary Video 3). This exceptional resilience, preserving the needle's physical form, highlights its potential as a next-generation reusable medical platform, likely for applications involving reloading or multi-stage use, while its stable structure also allows for single-step co-encapsulation.

Based on this advanced material platform, the therapeutic efficacy of H/E@ACA MNs stems from their multimodal attack on the core pathology of infected burn wounds. Traditional antimicrobial strategies are increasingly challenged by antibiotic resistance. To overcome this critical hurdle, our work pioneers a shift from conventional antibiotics to a targeted genetic antibiofilm weapon: AS*yycF*. The selection of this target was based on its well-established, critical biological function. The YycFG two-component system (TCS) is a known and highly conserved sensing pathway in many Gram-positive bacteria, which plays a pivotal regulatory role in cell wall homeostasis and biofilm formation 34. Given that *yycF* is the essential response regulator within this pathway, we rationally designed an ASO in our prior research to specifically silence *yycF* expression, hypothesizing this would be an effective strategy to disrupt biofilm integrity 35. Our current study confirms that ASO-mediated *yycF* silencing effectively disrupts bacterial biofilm integrity (Fig. 3). It is noteworthy that we employed this integrated platform rather than ASO monotherapy, as naked oligonucleotides are susceptible to rapid degradation and exhibit limited efficacy in complex wound environments without the protective delivery provided by the sIPN matrix 18, 26.

Simultaneously, the synergistic action of CeO_2_ and An directly counteracts the hostile microenvironment. Enzyme-mimicking CeO2 scavenges harmful H_2_O_2_ and generates vital O_2_ to alleviate tissue hypoxia (Fig. 2 L-M), while the potent natural compound An exerts powerful antioxidant and anti-inflammatory effects, mitigating oxidative damage and suppressing excessive inflammation 36, 37, widely reported in various inflammatory contexts 38-41. This decisive remodeling of the wound bed creates a pro-regenerative milieu, which was first observed in our *in vitro* results demonstrating a coordinated cellular response, including M2 macrophage polarization and enhanced angiogenesis (Fig. 3). This beneficial shift was faithfully recapitulated in our infected burn wound model. Histological analysis confirmed a decisive transition from an inflamed to a healing state, resulting in the accelerated deposition of high-quality, well-structured collagen. Notably, our data revealed a COL I/COL III ratio closer to that of healthy skin, along with the regeneration of skin appendages (Fig. 6), indicating that the H/E@ACA platform promotes true regeneration rather than mere fibrotic repair—a key step in preventing hypertrophic scarring 42.

To elucidate the molecular drivers of this pro-regenerative program, we performed transcriptome analysis, revealing significant upregulation of key developmental signaling pathways, most notably the Wnt pathway (Fig. 8 C and E). The Wnt signaling pathway is a cornerstone of skin morphogenesis and regenerative healing 43, 44. Our studies further confirmed this finding, identifying Krüppel-like factor 5 (KLF5) as a key downstream effector. KLF5 is considered a "master regulator" that regulates cell proliferation, stem cell maintenance, and epithelialization during tissue repair 45-47. Its expression is known to be tightly regulated by the Wnt/β-catenin signaling pathway 48, 49. Our findings establish a robust mechanistic cascade: H/E@ACA materials activate the Wnt pathway, which in turn upregulates the master regulator KLF5, orchestrating the complex cellular processes required for high-quality skin regeneration. This mechanism is not only correlative; it has been validated at the protein level and further explored using specific inhibitors. Targeted inhibition of KLF5 using ML264 successfully reversed its upregulation *in vivo* (Fig. 8K), providing strong evidence for a direct causal relationship. Collectively, our findings suggest a hierarchical regenerative mechanism: the multimodal dismantling of the hostile microenvironment, encompassing biofilm eradication, ROS scavenging, hypoxia alleviation, and immunomodulatory M2 polarization, acts as the upstream trigger, which creates a permissive niche to subsequently activate the Wnt/KLF5 axis as the downstream amplification pathway for high-quality tissue repair. To our knowledge, this is the first study to link multifunctional MN therapy to a specific mechanism of activation of the epidermal Wnt/KLF5 signaling axis in the context of infectious wound healing. However, we acknowledge that wound healing is a multifactorial process; while Wnt/KLF5 acts as a key driver here, other parallel pathways undoubtedly contribute to this regeneration.

While this study provides strong evidence in a rat model, we acknowledge its limitations. First, considering the anatomical differences between rat and human skin, future studies in large animal models are warranted to further validate clinical translatability. Second, although the 24-day endpoint demonstrated effective closure, longer-term observation is required to comprehensively evaluate scar maturation and the complete regeneration of skin appendages. Finally, this study focused on MRSA; exploring the platform's efficacy against Gram-negative pathogens such as P. aeruginosa remains an important direction for future research.

In summary, we developed an on-demand, mechanically robust sIPN microneedle platform that achieves comprehensive microenvironment remodeling via targeted gene silencing and multimodal microenvironment modulation. This platform systematically eradicates MRSA biofilms and neutralizes oxidative/hypoxic stress, which consequently triggers the epidermal Wnt/KLF5 regenerative axis, acting as a key driver within the multifactorial wound healing process. By breaking the pathological cycle of infected burns, this study provides a highly efficient and translatable strategy for high-quality skin regeneration.

## Supplementary Material

Supplementary figures and table, video legends, fact sheet.

## Data Availability

All other data are available from the corresponding authors upon request. Source data are provided with this paper.

## Abbreviations

An: anthocyanin
ASO: antisense oligonucleotide
BP: biological processes
CC: Cellular Component
CeO₂: nanoceria
DEGs: differentially expressed genes
ECM: extracellular matrix
ETPTA: ethoxylated trimethylolpropane triacrylate
HAMA: hyaluronic acid methacrylate
KLF5: Krüppel-like factor 5
MF: molecular function
MN: microneedle
MRSA: Methicillin-resistant Staphylococcus aureus
PDMS: polydimethylsiloxane
ROS: reactive oxygen species
SD: standard deviation
sIPN: semi-Interpenetrating Polymer Network
TCS: two-component system
