# Supplementary material for: On-demand sIPN microneedles promote infected burn wound healing via microenvironment remodeling and activation of the Wnt–KLF5 regenerative axis
Source: Theranostics. 2026 May 1;16(12):6509–28. doi: 10.7150/thno.130253 (PMC13231964; doi:10.7150/thno.130253)
Supplement: Supplementary file 1 — Supplementary figures and table, video legends, fact sheet. [file thnov16p6509s1.zip › Supplementary Materials.pdf]

SUPPLEMENTARY

Supplementary Information

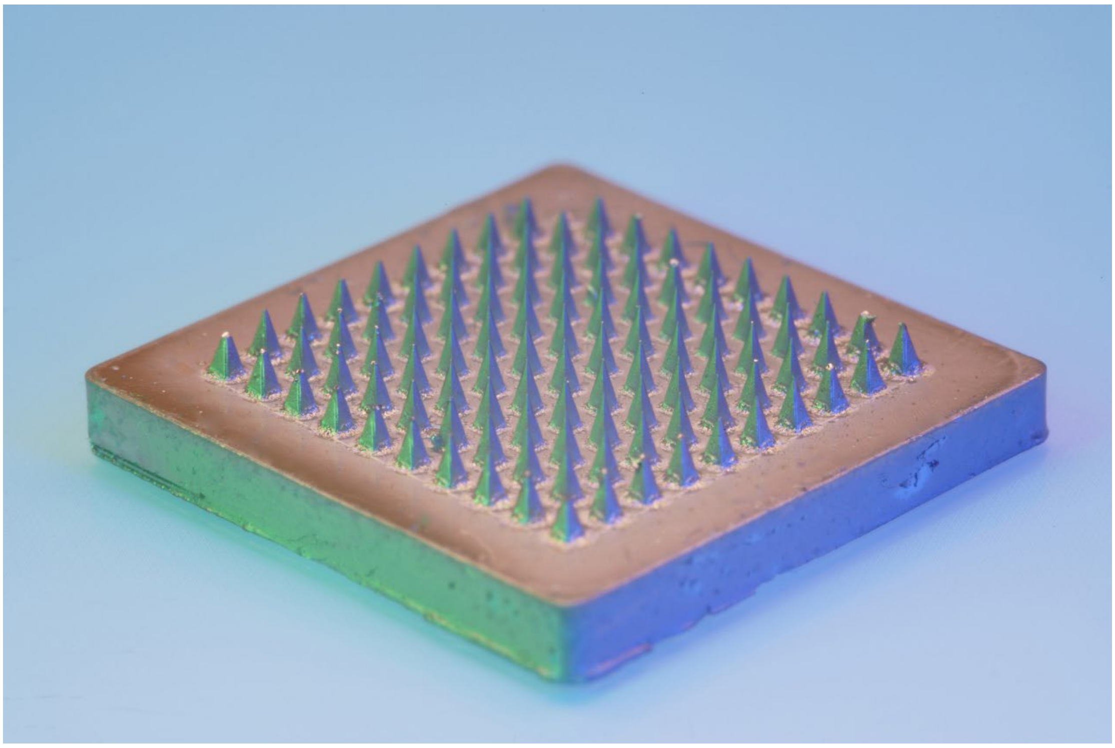

Figure S1: Macrophotograph of a Gold-Sputtered Microneedle under Controlled Illumination. A macroscopic photograph of a representative microneedle patch prepared for SEM imaging. The image highlights the exceptional structural integrity and uniformity of the needles, with sharp tips and consistent geometry across the array. This high-fidelity replication of the mold features underscores the high success rate and precision of our on-demand fabrication method.

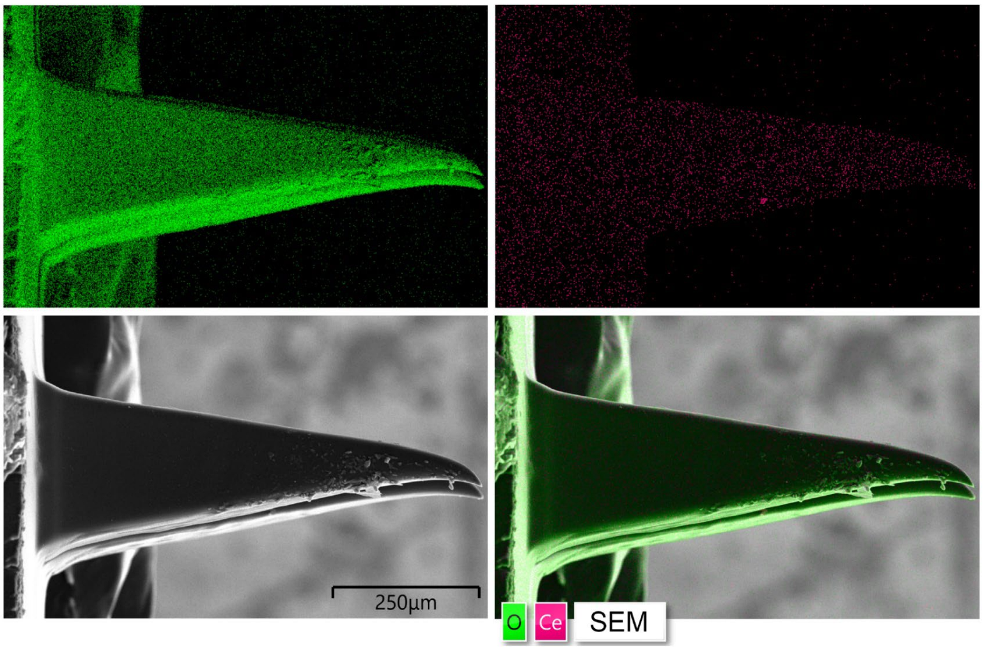

Figure S2: Elemental distribution of the MNs. Representative SEM image (bottom left) and corresponding EDS elemental mapping images of a single microneedle, revealing the spatial distribution of Oxygen (green, top left) and Cerium (magenta, top right). The merged image (bottom right) confirms the homogeneous incorporation of cerium-containing payloads throughout the needle tip. Scale bar = 250 μm.

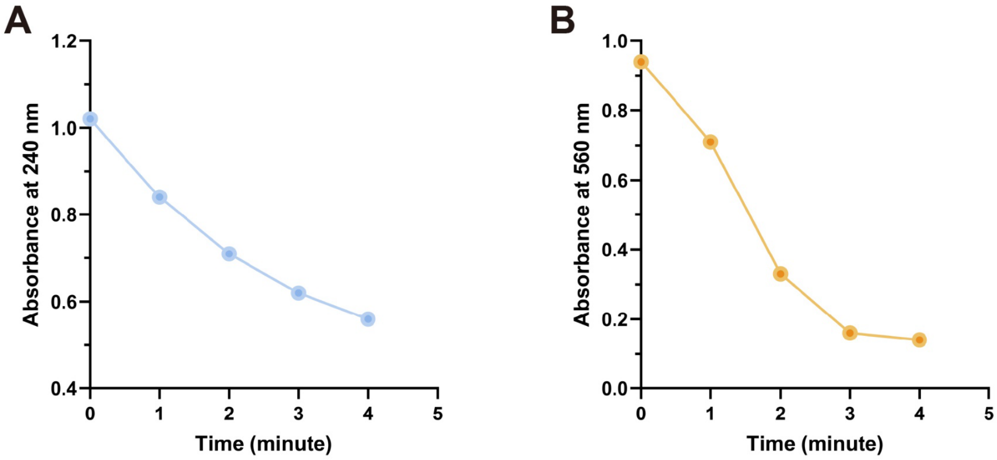

Figure S3: Characterization of enzyme-mimicking activities. (A, B) Quantitative analysis of the catalytic performance: (A) CAT-mimicking activity showing the reduction of H_2O_2 absorbance at 240 nm over 4 minutes, and (B) SOD-mimicking activity showing the reduction of superoxide radical absorbance at 560 nm over 4 minutes.

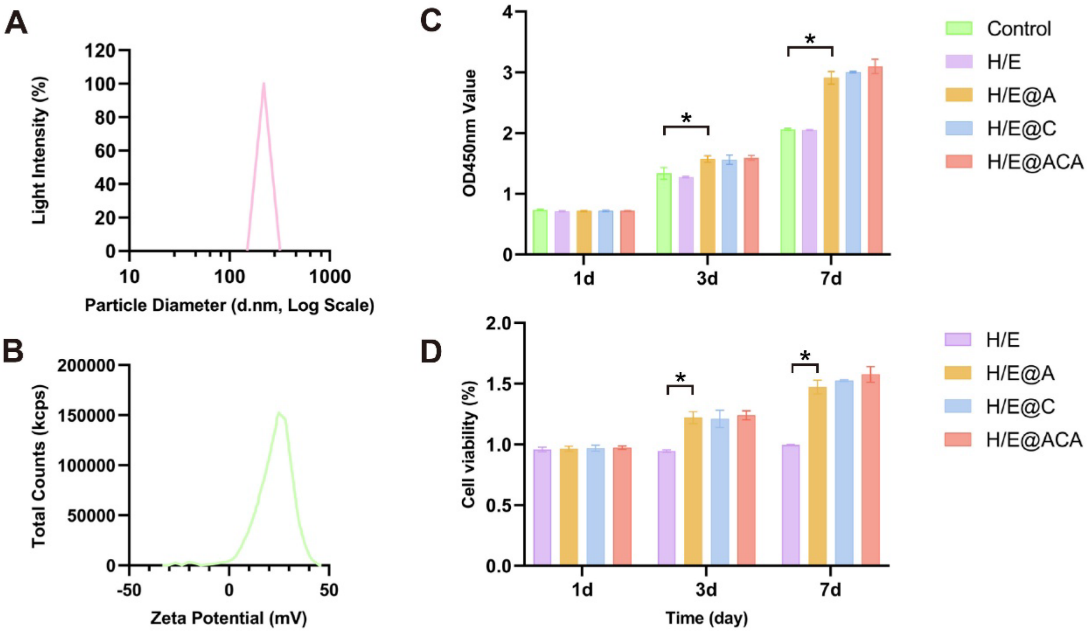

Supplementary Fig. S2: Characterization of CeO2 and Quantitative analysis of cell viability. (A) particle size distribution and (B) zeta potential. (C, D) Quantitative analysis of cell viability at day 1, 3, and 7. *, $p < 0.05$.

Figure S4: Characterization of CeO2 and Quantitative analysis of cell viability. (A) particle size distribution and (B) zeta potential. (C, D) Quantitative analysis of cell viability at day 1, 3, and 7. *, $p < 0.05$.

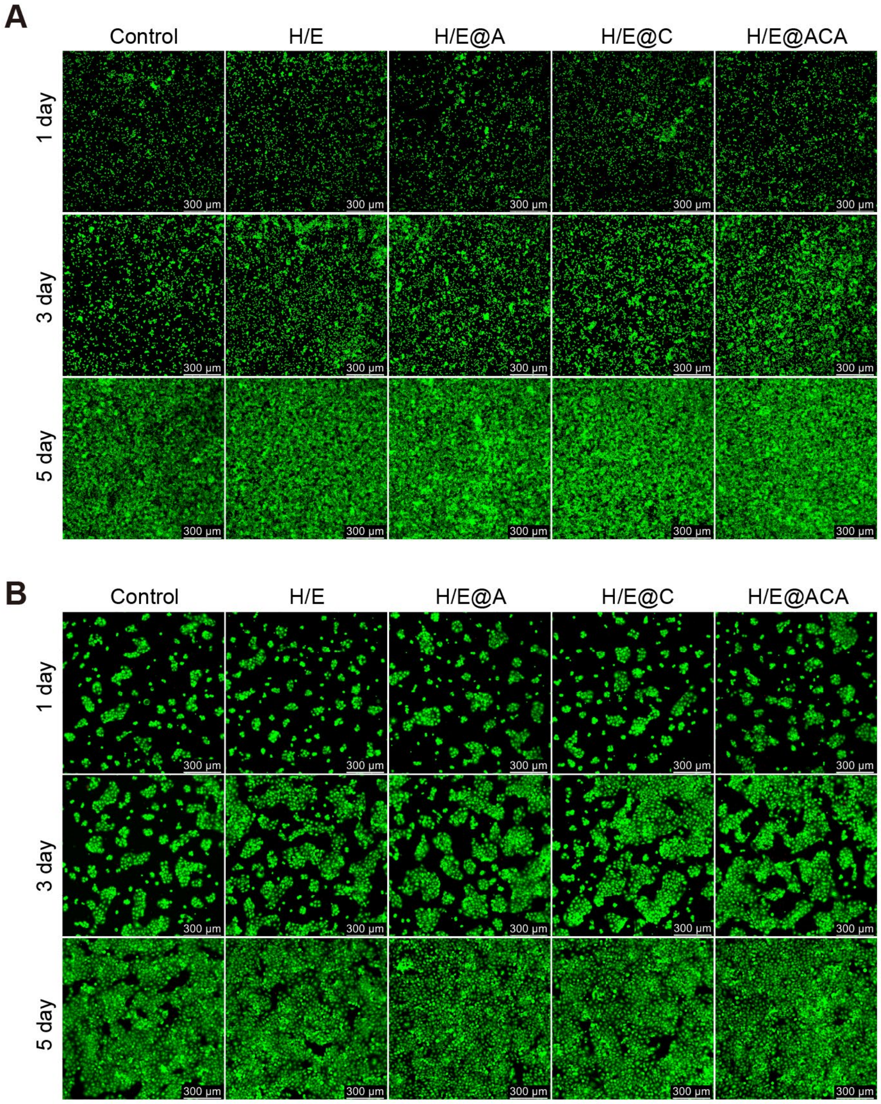

Figure S5: Representative fluorescence images of Live/Dead staining of RAW 264.7 macrophages (A) and HaCaT keratinocytes (B) cultured with MNs extracts. Scale bar = 300 μm.

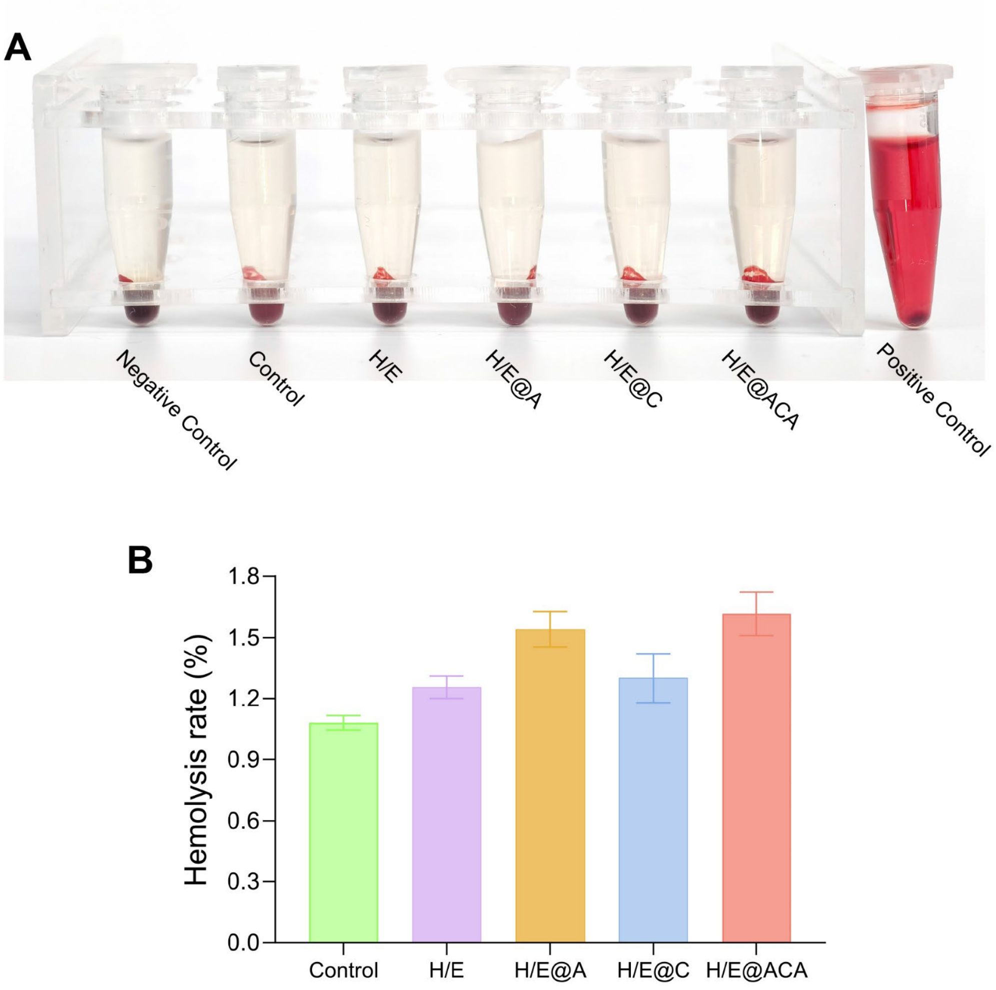

Figure S6: Hemocompatibility evaluation of the MNs. (A, B) In vitro hemolysis assay of red blood cells treated with different samples: (A) representative photographs of the supernatant after centrifugation, where water and PBS were used as positive and negative controls, respectively; and (B) quantitative analysis of the hemolysis rates based on absorbance readings. Data are presented as mean ± SD (n = 3).

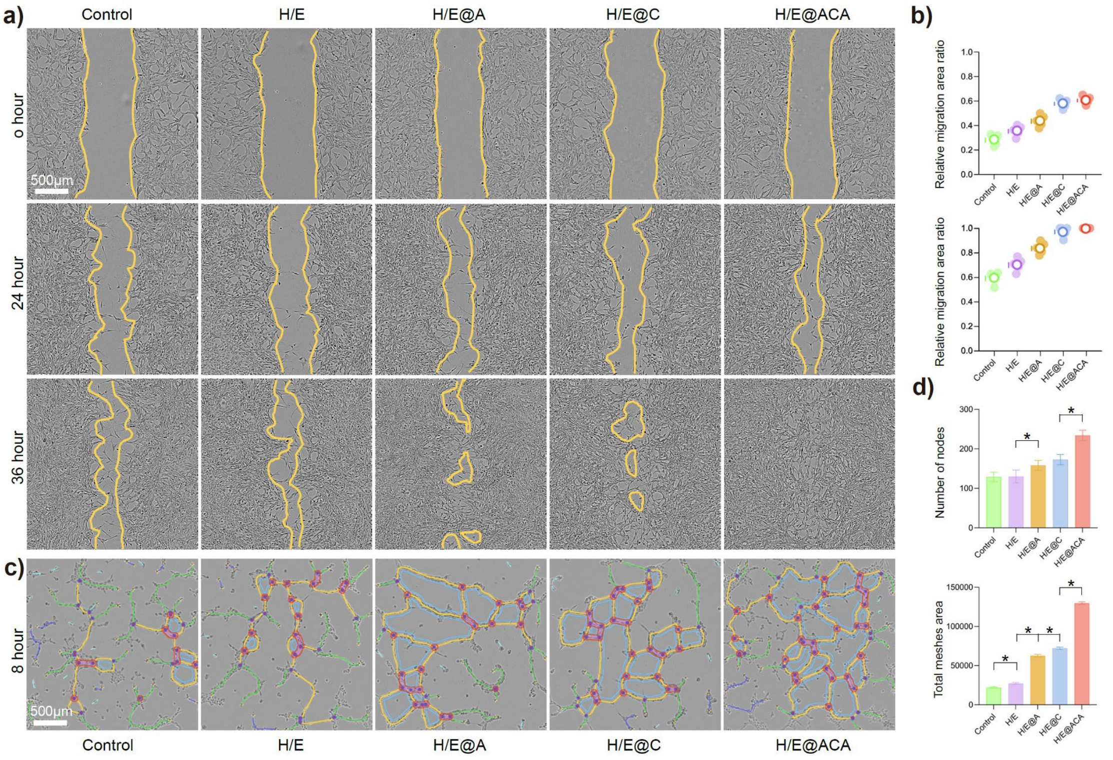

Figure S7: Promotion of Cell Migration and Tube Formation. (A, B) Scratch wound healing assay with HUVECs cultured in different MNs extracts: (A) representative images at different time points and (B) semi-quantitative analysis of relative migration area. Scale bar = 500 µm. (C, D) Tube formation assay with HUVECs cultured in different MNs extracts: (C) representative images and (D) corresponding quantitative analysis of the number of nodes and total mesh area. Scale bar = 500 µm. *, $p < 0.05$.

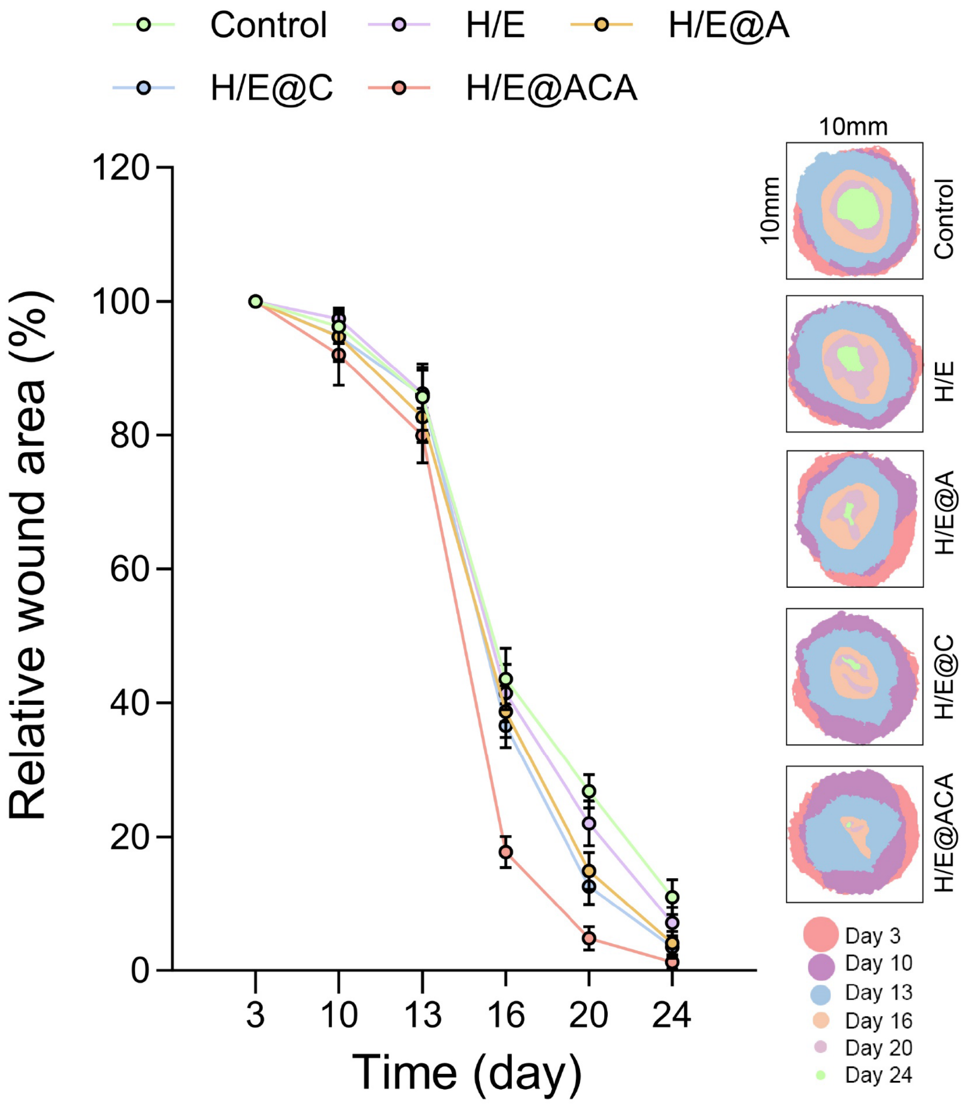

Figure S8: Quantitative analysis of in vivo wound closure. Evaluation of the wound healing process in a rat model of MRSA-infected full-thickness burns over 24 days. The quantitative curves of relative wound area percentage versus time (left) demonstrate a significantly accelerated healing rate in the H/E@ACA group compared to the control and other treatment groups. This trend is visually corroborated by the representative superimposed wound traces (right), which depict the progressive centripetal contraction of the wound margins from Day 3 to Day 24. Data are presented as mean ± SD (n = 5).

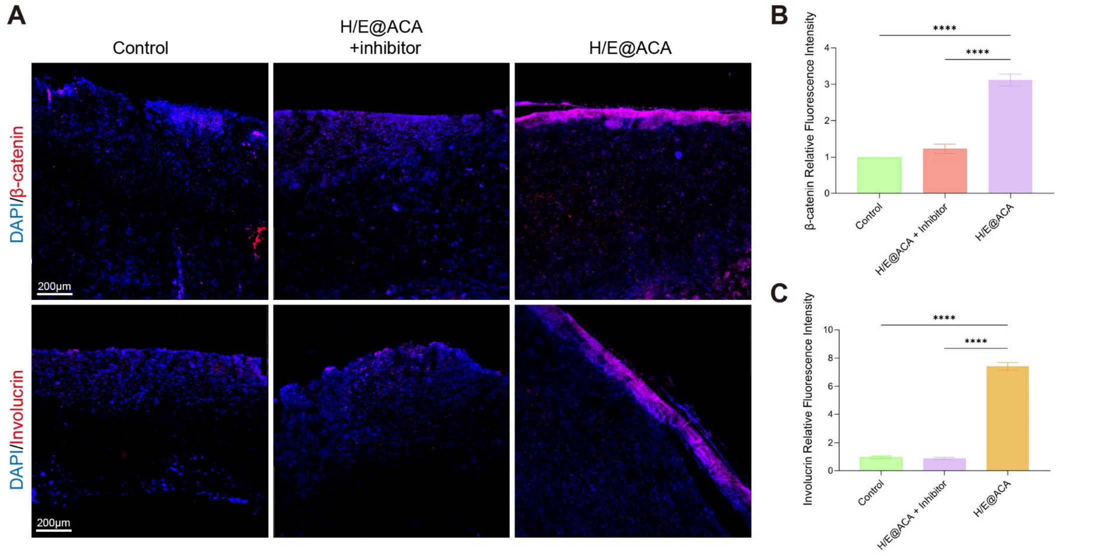

Figure S9: Immunofluorescence validation of Wnt/KLF5-mediated epidermal regeneration. (A) Representative immunofluorescence images of β-catenin (red) and Involucrin (red) in rat wound tissues. The H/E@ACA group shows robust, spatially defined accumulation of β-catenin and Involucrin specifically within the regenerating epidermis, indicating activation of the Wnt pathway and proper epidermal stratification. Co-treatment with the Wnt inhibitor XAV-939 significantly abolished these effects. Scale bar = 200 μm. **(B, C)** Quantitative analysis of relative fluorescence intensity for β-catenin **(B)** and Involucrin **(C)**, confirming that blockade of Wnt signaling suppresses the expression of regeneration markers. Data are presented as mean ± SD (n = 3). ****p < 0.0001.

Table S1. Comparison of Preparation Time for Photopolymerized Hydrogel Microneedles

Photopolymerization system	Critical process	Waiting time before demolding	The total preparation time
GelMA MN[1]	The precursor was centrifuged for 5 min, and UV-cured for 10–300 s.	Remove the mold after 24 hours of exposure to light and in a dry environment	About 24 h
GelMA/PEGDA MN[2]	The precursor was dissolved at 50 °C, degassed by vacuum or centrifugation, and then UV-cured.	Remove the mold after 24 hours of exposure to light and in a dry environment.	About 24 h
MeHA MN[3]	The filled molds were centrifuged at 4000 rpm for 5 min, dried overnight in a fume hood, UV-crosslinked for 3–15 min, and then demolded.	Overnight drying	About 12–24 h
HAMA/PVA MN[4]	After molding, the mixture was vacuum-degassed and dried at room temperature for 4 h, the needle tips were cured with 405-nm blue light for 20 s, a PVA base was added and dried for 12 h, and the MNs were then demolded.	4 hours + 12 hours staged drying	About 16 h
HAMA/ETPTA MN	After molding with centrifugal tip filling, the MNs were polymerized under UV for 60s, immediately demolded, and the solvent was evaporated at 37 °C for 90 min.	90 minutes of solvent evaporation	About 90 min

1 Zhu, J. *et al.* Gelatin Methacryloyl Microneedle Patches for Minimally Invasive Extraction of Skin Interstitial Fluid. *Small* **16**, e1905910, doi:10.1002/smll.201905910 (2020).

2 Yuan, M. *et al.* GelMA/PEGDA microneedles patch loaded with HUVECs-derived exosomes and Tazarotene promote diabetic wound healing. *J Nanobiotechnology* **20**, 147, doi:10.1186/s12951-022-01354-4 (2022).

3 Chew, S. W. T. *et al.* A self-adhesive microneedle patch with drug loading capability through swelling effect. *Bioeng Transl Med* **5**, e10157, doi:10.1002/btm2.10157 (2020).

4 He, C. *et al.* Microneedles combining delivery of hUMSC-derived exosomes and EGCG mitigate UV-induced skin damage. *J Nanobiotechnology* **23**, 643, doi:10.1186/s12951-025-03735-x (2025).

Supplementary Video 1: Demonstration of the rapid, high-fidelity demolding of the microneedle patch immediately after photopolymerization. This video shows the H/E@ACA microneedle patch being demolded from its PDMS mold immediately after a 60-second UV photopolymerization. The inclusion of ETPTA provides instant mechanical integrity, enabling immediate, high-fidelity demolding with a high success rate. This process eliminates the prolonged in-mold drying (24-48 hours) required by conventional methods, facilitating a true on-demand fabrication workflow.

Supplementary Video 2: Demonstration of the strong tissue adhesion and grip of the microneedle patch on the infected burn rat model. To showcase its stability, the patch is subjected to significant external forces. As shown, the patch remains firmly anchored to the wound surface without delamination or detachment. This robust grip is crucial for ensuring stable, long-term adhesion in dynamic physiological conditions, which is essential for effective drug delivery and wound healing applications.

Supplementary Video 3: Long-term Stability and Dynamic Structural Reset. This clip features used microneedles (MNs) that were retrieved after a six-month immersion in either culture medium or PBS. Following a one-hour drying period at 37°C, they undergo rapid deswelling and revert to their initial, well-preserved morphology and size—a process akin to a 'factory reset'. This characteristic, which we term 'Dynamic Structural Reset', not only demonstrates their outstanding durability but also unlocks immense commercial potential for them to serve as a next-generation reusable medical platform.

Manufacturer's comprehensive technical data sheet about CeO_2

江苏先丰纳米材料科技有限公司

XFI66-介孔二氧化铈纳米颗粒

XFI66- Mesoporous ceria nanoparticles

1　技术参数(Properties)

名称	**Product name**	介孔二氧化铈纳米颗粒<br>Mesoporous ceria nanoparticles
浓度	**Concentration**	1mg/mL
颗粒尺寸	**Particle size**	80-105 nm （TEM）
外形	**Appearance**	乳白色溶液 Milk white dispersion
电位	**Zeta potential**	~5 mV

备注：电位数据为单次测量数据，不同批次之间允许浮动。

Note: Zeta potential data is single measurement and allowed to float between different batches.

2　表征测试图(Characterizations)

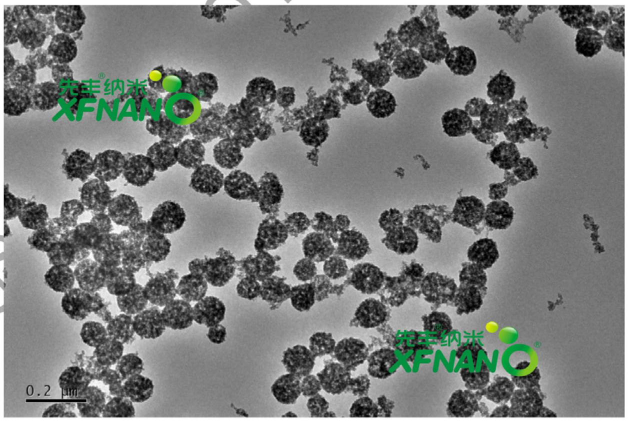

图 1

Jiangsu XFNANO Materials Co.,Ltd

江苏先丰纳米材料科技有限公司
地址：江苏省南京市浦口区步月路 29 号紫峰研创中心一期 9 栋
邮编：211806
电话 **Tel**：400-025-3200(免长途费)
传真 **Fax**：025-68256991

Jiangsu XFNANO Materials Tech. Co.,Ltd
Add：Nanjing City, Jiangsu Province, China
Zip：for 211806
E-mail：sale@xfnano.com
http://www.xfnano.com

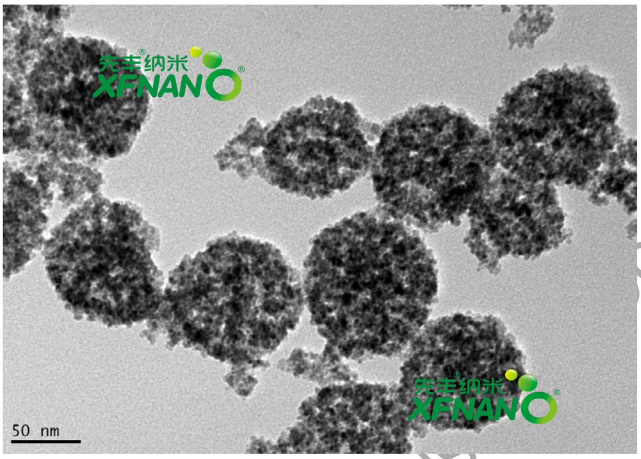

图 2

图 1，2　介孔二氧化铈纳米颗粒 TEM 图

Fig.1,2　TEM images of Mesoporous ceria nanoparticles

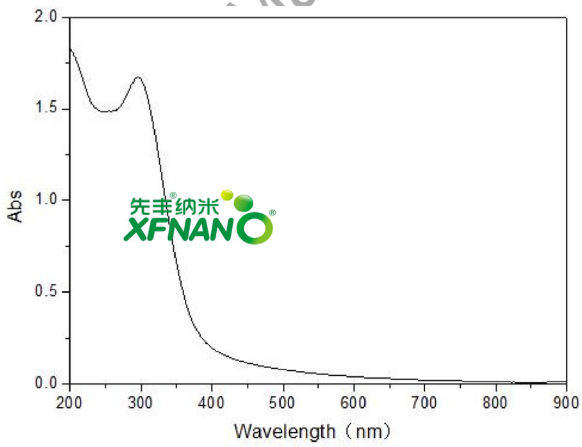

图 3　介孔二氧化铈纳米颗粒的 UV-vis 图（<1mg/mL）

Fig.3　UV-vis diagram of Mesoporous ceria nanoparticles（<1mg/mL）

Jiangsu XFNANO Materials Tech. Co.,Ltd

3 应用领域(Application Fields)

二氧化铈纳米颗粒具有广泛的抗氧化活性，可清除自由基。具有模拟酶活性的催化作用，如：超氧化物歧化酶（SOD）、过氧化氢酶等。

Ceriumdioxide nanocubes have extensive antioxidant activity and can scavenge free radicals. It has The catalytic effect of mimicking enzyme activity, such as:super oxide dismutase (SOD), catalase, etc.

4 储存条件(Storage Conditions)

4℃冷藏避光密封保存，切勿冻存。保存期限：3 个月。

Sealed, avoidlight, and keep at 4℃. Donot freeze. Expiry date: 3months.

5 先丰寄语（Messages from XFNANO）

二氧化铈纳米颗粒粒径均一，单分散性较好，易于表面功能化。另外，二氧化铈纳米颗粒生物毒性低，可用于下游细胞动物实验。

Ceria nanoparticles have a uniform particle size, good monodispersity, and easy surface functionalization. In addition, ceria nanoparticles have low biotoxicity and can be used in downstream cell animal experiments.

声明：我司保证技术报告中的信息的准确性，但不保证材料的普适性，也不负责由此导致的任何损失。提供的参数为统计数据，允许少量浮动。且我司提供的技术报告都是面向所有客户，报告中的表征图片请勿擅自截图使用。

Disclaimer: XFNANO LLC believes that the information in this Technical Data Sheet is accurate and represents the best and most current information available to us. XFNANO Material makes no representations or warranties either express or implied, regarding the suitability of the material for any purpose or the accuracy of the information contained within this document. Accordingly, XFNANO Material will not be responsible for damages resulting from use of or reliance upon this information. Please do not use screenshots of any characteristic graphs in this report without permission.

Jiangsu XFNANO Materials Tech Co.,Ltd

2023.11.22 Ed.1

江苏先丰纳米材料科技有限公司
地址：江苏省南京市浦口区步月路 29 号紫峰研创中心一期 9 栋
邮编：211806
电话 **Tel:** 400-025-3200(免长途费)
传真 **Fax:** 025-68256991

Jiangsu XFNANO Materials Tech. Co.,Ltd
Add: Nanjing City, Jiangsu Province, China
Zip: for 211806
E-mail: sale@xfnano.com
http://www.xfnano.com
